# The interplay of antiaromaticity and diradical character in diarenoindacenes and diindenoarenes

**DOI:** 10.1039/d5sc05050b

**Published:** 2025-10-02

**Authors:** Efrain Vidal, Gabrielle I. Warren, Joshua E. Barker, Michael M. Haley

**Affiliations:** a Department of Chemistry & Biochemistry and the Materials Science Institute, University of Oregon Eugene OR 97403-1253 USA haley@uoregon.edu

## Abstract

Over the past ∼15 years our group has performed multiple structure/properties relationship studies to assess how logical structural refinement can affect the antiaromaticity/diradicaloid continuum. Using precision organic synthesis, we can alter the chemical composition of both the pro-aromatic core and the outer fused arene groups. The rational design of antiaromatic diareno-fused *s*-indacene derivatives leads to pronounced variation of molecule paratropicity, *i.e.*, the HOMO–LUMO energy gap, as determined experimentally (NMR, CV, UV-Vis, X-ray data) and computationally (NICS-XY scans, NICS2BC, bond current plots). Successive benzinterposition within the core motif affords diindenoarene structures where compound paratropicity is minimized yet diradicaloid character emerges. Using the same techniques of changing outer ring fusion with aromatic carbocycles and heterocycles created a series of structures where the diradical character and thus the singlet–triplet energy gap of the molecule could be varied in a controlled, predictable manner, as determined experimentally (NMR, CV, UV-Vis, X-ray, SQUID data) and computationally using high-level quantum chemical calculations. Arising from these fundamental studies, we have demonstrated that diarenoindacenes and diindenoarenes can act as the active layer in OFETs, often showing ambipolar charge characteristics with hole mobilities as high as 7 cm^2^ V^−1^ s^−1^. We also established that our quinoidal/diradicaloid compounds possess large, atypical anti-ohmic conductance enhancement of their transport properties at longer molecular lengths, which suggests that this class of organic materials is a promising candidate for creating highly conductive and tunable nanoscale wires. Taken as a whole, our studies show that traditional physical organic chemistry concepts can be readily applied to modern organic materials research.

## Abstract 2

This perspective describes our efforts on how to rationally alter the electronic and magnetic properties of a class of inter-related quinoidal/diradicaloid molecules by judicious structural modification.

## Graphical abstract

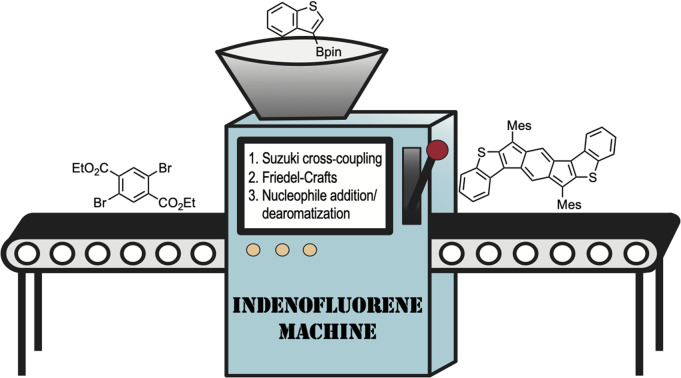

## Introduction

Aromaticity and antiaromaticity are fundamental concepts in organic chemistry that still sustain considerable discussion and research within the community.^1^ The [4*n* + 2] π-electron rule, proposed by Hückel in 1931 and codified by Doering in 1951,^2^ continues to be taught and permits quick identification of aromatic species. Breslow and Dewar later postulated the [4*n*] π-electron rule for systems which they coined the term “antiaromatic”.^3^ Antiaromaticity is the “opposite” of aromaticity, in that the ring current (in the presence of a magnetic field) is diatropic (stabilizing) for aromatic molecules and paratropic (destabilizing) for antiaromatic molecules. Antiaromatic molecules are of fundamental interest because of their extremely reactive nature, making them nearly impossible to isolate in their unsubstituted form.^4^ As a result, antiaromatic molecules find pathways to relieve this destabilization. For example, cyclooctatetraene (COT) adopts its well-known, nonplanar tub shape, and cyclobutadiene undergoes rapid dimerization.

Polycyclic antiaromatic hydrocarbons (PAAHs), compounds that contain one or more antiaromatic motifs as part of the overall hydrocarbon backbone, tend to be the black sheep of π-conjugated molecules and materials. Unlike the well-studied polycyclic aromatic hydrocarbons (PAHs) that often feature multiple stabilizing, aromatic [4*n* + 2] π-electron pathways,^4,5^ inclusion of the antiaromatic [4*n*] π-electron unit(s) very often results in significant destabilization of the resultant molecule. Nonetheless, interest in PAAHs has seen a strong resurgence over the last ∼15 years because of the promising optoelectronic properties that are inherent to these compounds, such as small HOMO/LUMO energy gaps, low HOMO and LUMO energy levels, redox amphoterism, and increased conductance;^6^ thus, such molecules could potentially find use in device applications such as organic field-effect transistors (OFETs) or organic solar cells (OSCs).

**Fig. 1:**
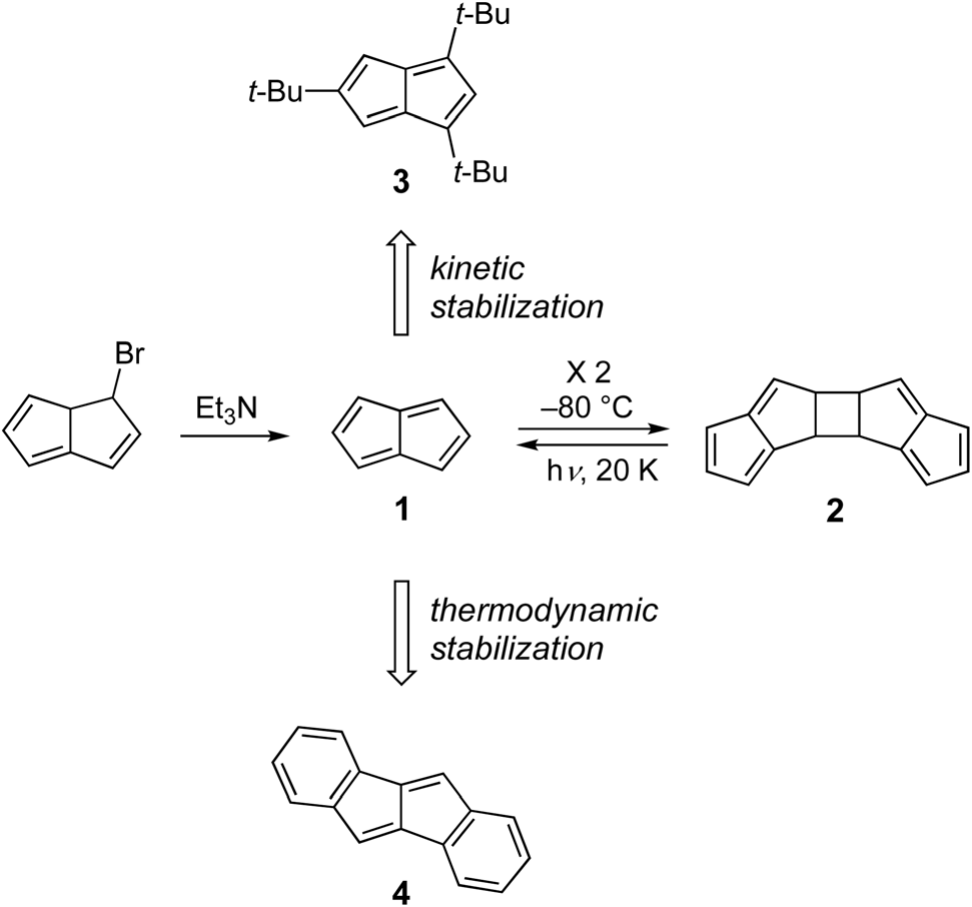
Two popular methods to tame antiaromatic molecules.

Fortunately, chemists have devised ways to moderate highly reactive antiaromatic molecules. For example, when Hafner attempted in the early 1970s to prepare the parent pentalene 1 (Fig. 1), a bicyclic 8 π-electron analogue of COT, the molecule rapidly dimerized into 2 even at −80 °C to alleviate the destabilizing paratropic ring current.^7^ Bally *et al.* showed that this process, which is thought to occur *via* diradical intermediates and thus indicative of the presence of a low-lying triplet state, is reversible upon photolysis of 2 in an Ar matrix, with the photocleavage occurring in two distinct steps—formation first of a diradical then regeneration of 1.^8^ To obtain an isolable hydrocarbon, Hafner subsequently installed three *t*-butyl groups onto the pentalene skeleton, *e.g.*, 3, thus kinetically stabilizing the molecule.^9^ Kinetic stabilization with bulky substituents was a very popular method in the 1970s and 1980s to generate a variety of stable, isolable antiaromatic compounds, including those with cyclobutadiene and indacene cores.^4^

Another widely-used strategy to tame paratropic compounds is to fuse one or more aromatic rings, such as in dibenzopentalene 4, a PAAH first reported in 1912, as such fusion results in a thermodynamically more stable hydrocarbon.^10^ This stabilization comes with a cost, however, as arene fusion weakens the paratropic ring current significantly, which results in an increased HOMO/LUMO energy gap and higher HOMO and LUMO energy levels compared to the parent antiaromatic hydrocarbon. Nonetheless, Kawase and Takimiya showed in 2010 that derivatized dibenzopentalenes could be used as an active layer in OFETs that showed modest hole mobilities (1.8 × 10^−3^ cm^2^ V^−1^ s^−1^) as well as be the electron-donor layer in OSCs (PCE = 0.94%), clearly demonstrating the materials potential of PAAHs.^11^ Very recently, a combination of steric blocking and extended conjugation with pendant aryl groups led to the isolation and thorough characterization of the 1,3,4,6-tetraphenyl derivative of 1.^12^

Properties associated with antiaromaticity drive motivation to study these systems in their own right, but there is a unique relationship between the antiaromatic nature of a molecule and irregular electronic configurations that bolsters the merit of investigating such species. A recent theoretical survey of antiaromatic [4*n*]annulenes conducted by Quintero *et al.* examined the role of frontier orbitals in a balancing act of stabilization from disjoint orbital character and inter- *vs.* intra-orbital electron–electron repulsions that favor unpaired electrons to varying degrees.^13^ For example, computationally forcing COT into a planar conformation permits investigation of this interplay between paired and unpaired configurations. The electronic structure of planar D_8h_ COT features unpaired spins residing in two degenerate singly-occupied molecular orbitals (SOMOs), which are stabilized by the non-bonding and disjoint character of the frontier orbitals. The authors describe D_8h_ COT as having an open-shell (OS) electronic configuration as opposed to one that is closed-shell (CS) based on these results. As this type of electronic stabilization can be extrapolated to other [4*n*]annulenes and their congeners *via* perturbation, antiaromatic molecules can be thought of as having some inherent diradical character on account of their frontier orbitals and configurational mixings.^13^

**Fig. 2:**
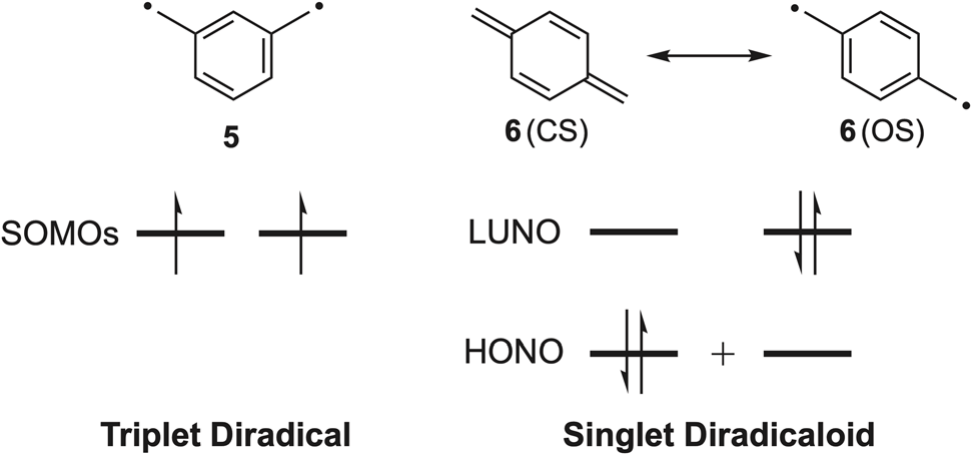
Depiction of frontier molecular orbitals for diradical 5 (two degenerate orbitals) and diradicaloid 6 (two nearly degenerate orbitals).

It is crucial to be clear and consistent regarding the description of diradical-like species. We will use the definition found in the IUPAC “Gold Book” for diradicals, which states that diradicals are “molecular species having two unpaired electrons, in which at least two different electronic states with different multiplicities [electron-paired (singlet state) or electron-unpaired (triplet state)] can be identified”.^14^ Pure diradicals are described as molecules in which two electrons occupy two degenerate non-bonding molecular orbitals (*e.g.*, *m*-xylylene/*m*-quinodimethane 5, Fig. 2), while the term diradicaloids is used to identify diradical-like molecules in which the two molecular orbitals are nearly degenerate (*e.g.*, *p*-xylylene/*p*-quinodimethane 6). Quantum chemical calculations are indispensable to any study of diradicaloids for determining the effects responsible for changes in diradical character. Standard diradical calculations involve determination of the diradical index value (*y*_0_), which represents the occupation of the lowest unoccupied natural orbital (LUNO) according to natural orbital occupation number (NOON) analysis. In this scheme, *y*_0_ = 1 represents pure OS character while *y*_0_ = 0 represents a pure CS system.^15^ The magnitude of the value is used as an indicator for the likelihood that a molecule will exhibit diradical-like properties, and compounds with intermediate *y*_0_ values are predicted to be diradicaloids. It is important to note that *y*_0_ value calculations depend heavily on functional selection and as a result they are only reliably compared when the functional and basis set are the same. This theoretical analysis of novel OS π-systems provides an additional handle for analyzing their utility in organic electronic materials as they share similar properties with CS antiaromatics (*e.g.*, narrow HOMO/LUMO energy gaps and amphoteric redox behavior). This behavior is exemplified in bisphenalenyl diradicaloids that display both hole and electron mobilities up to 3 × 10^−3^ cm^2^ V^−1^ s^−1^.^16^

**Fig. 3:**
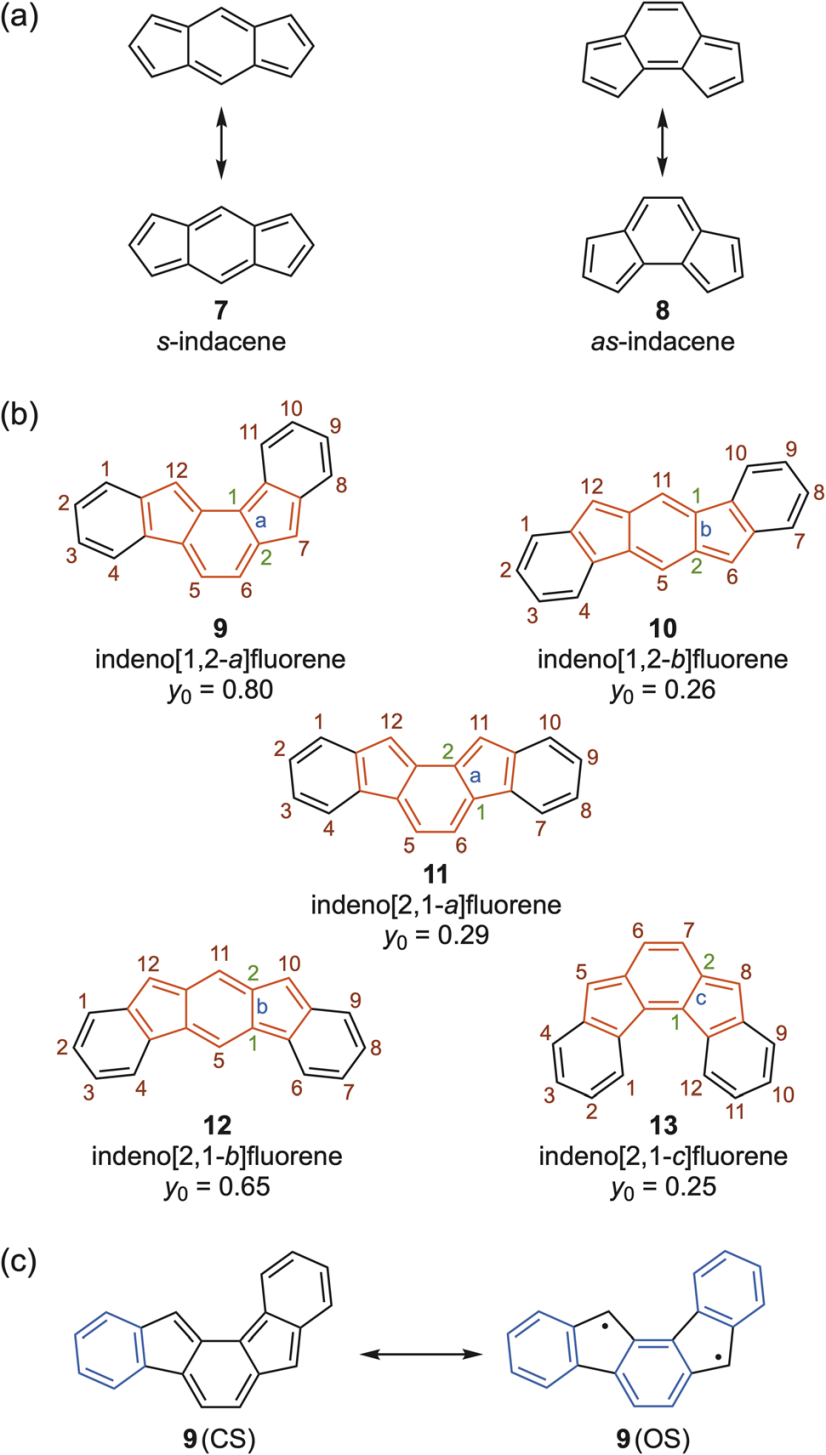
Isomeric (a) indacenes 7–8 and (b) indenofluorenes 9–13; the indacene core is highlighted in red in the latter examples. The computed values of *y*_0_ for the parent hydrocarbons are taken from ref. 26. (c) Closed-shell (CS) and open shell (OS) resonance forms of IF 9.

Installing one six-membered ring between the two five-membered rings of pentalene, a process long-ago called “benzinterposition”,^17^ affords the tricyclic hydrocarbon known as indacene. Depending upon the symmetric or asymmetric fusion of the five-membered rings on the six-membered ring leads to *s*-indacene 7 and *as*-indacene 8 (Fig. 3a).††As one reviewer noted, the name *as*-indacene is not entirely accurate, as “asymmetric” 8 does possess a mirror plane of symmetry. While the names linear-indacene and angular-indacene might be more descriptive for 7 and 8, respectively, we see no reason to change long-established literature precedent.^4,18^ As one might expect, Hafner and co-workers investigated antiaromatic 7 as well, finding that inclusion of four *t*-butyl groups kinetically stabilized^19^ the otherwise highly reactive molecule.^20^ A number of similarly stabilized *s*-indacenes have been disclosed over the subsequent ∼40 years.^21^ Very recent work by Tobe and co-workers showed that a series of stable hexaaryl-substituted derivatives of 7 could be readily prepared and their properties modulated by the aryl substituents.^22^ Interestingly, even though the *as*-indacene motif has been incorporated into pentacyclic systems and even larger structures (*vide infra*), studies on 8 or simple tricyclic derivatives thereof have not been reported in the literature.

In fall 2009, the Haley group initiated studies into indacene derivatives. Similar to Kawase and others, we elected to include benzo-fusion to help stabilize the paratropic core. Rather than “dibenzoindacene” or “diindenobenzene”, IUPAC nomenclature calls such structures indenofluorenes (IFs), of which there are five possible regioisomers (9–13) depending upon whether the structure contains an *s*- or *as*-indacene core, shown in red in Fig. 3b. When we started our experiments, only two of the five IF isomers had been described: LeBerre reported in 1957 the attempted synthesis of the 11,12-diphenyl derivative of 11, which proved to be extremely unstable.^23^ In 1994 the Swager group disclosed the 5,6,11,12-tetraiodo analogue of 10 that also was prone to rapid decomposition.^24^ Two years later, Scherf and co-workers claimed the synthesis of 6,12-diphenyl analogue of 10 as a model system for an indenofluorene-based polymer;^25^ however, the paper included no experimental or characterization data to corroborate this structure aside from an absorption spectrum *λ*_max_ of 543 nm.‡‡Before modern journal requirements, SI documents as part of a publication were a rarity; thus, ref. 25 had no SI file describing the properties or spectral data of 6,12-diphenylindeno[1,2-*b*]fluorene. Prof. Scherf kindly sent the corresponding author pages from the student's PhD thesis about this molecule, which showed that the data in the thesis matched identically to those reported in ref. 42. As of late 2009, there was no definitive evidence of stable, well-characterized, fully-conjugated indenofluorenes in the literature.

The calculated *y*_0_ values of the IF regioisomers (Fig. 3b) offer some insight as to why LeBerre and Swager had difficulty with their systems, as all IF isomers are predicted to possess modest (10, 11, 13) to pronounced (9, 12) diradicaloid character.^26^ This is especially easy to understand for molecules 9 and 12, as in the open shell form there are three aromatic Clar sextets *versus* only one sextet in the closed shell form (shown in blue in Fig. 3c), in addition to the contribution of 5 in the open-shell form of 9 and 12. For compounds 10, 11, and 13, the closed shell form possesses two Clar sextets as well as the presence of an *ortho*- or *para*-quinodimethane core, which results in an appreciable drop in diradical character. Unfortunately, any regain of aromatic stabilization is offset by the introduction of radical reactivity at the apical carbons of the five-membered rings. These positions too can be kinetically stabilized by the introduction of bulky aryl groups (*e.g.*, mesityl) on the apical carbon atoms, *e.g.*, positions 6/12 in 10. Tobe and co-workers prepared 10,12-dimesitylindeno[2,1-*b*]fluorene in 2013 and confirmed *via* multiple techniques that the molecule, while possessing a singlet diradicaloid ground state, exhibited significant triplet character at room temperature.^27^ Haley and co-workers found in 2017 that the 7,12-dimesityl derivative of 9, predicted to be a ground state triplet, was so reactive that the molecule had to be analyzed in a dilute, degassed solution. While the obtained spectral data implicated formation of the [1,2-*a*]IF, confirmation of its successful synthesis came about with the X-ray crystal structure of the dianion reduction product, confirming the correct skeletal connectivity.^26^ Until 2024 (*vide infra*), these were the only examples of the [1,2-*a*]IF and [2,1-*b*]IF isomers, so they will not be discussed further. The Tobe and Haley groups reported the syntheses of stable, isolable derivatives of the [2,1-*a*]IF^28^ and [2,1-*c*]IF^29^ regioisomers in 2011 and 2013, respectively, of which the spectral (NMR, UV-Vis) and structural (X-ray) data support the dominance of the closed-shell structure. Both isomers have been revisited by the Haley^30^ and Das^31^ groups in recent years; still, the number of [2,1-*a*] and [2,1-*c*]IFs is rather limited, so these too will not be discussed further. By far, the [1,2-*b*]IF scaffold, and its structurally-related, heterocycle-fused *s*-indacene congeners (*vide infra*), will be the main focus of the antiaromaticity studies described in this article. We refer the reader to several reviews with more information on the other IF regioisomers.^18,32^

### Synthetic strategies

Our studies of PAAHs began in fall 2009 as a rotation project for first-year graduate student Brad Rose. We repeated the synthesis of the 5,6,11,12-tetraiodo analogue of 10*via* double transannular cyclization of octadehydrodibenzo[12]annulene;^24^ however, all attempts at Sonogashira cross-coupling degraded the IF starting material. Rather, we introduced the four (triisopropylsilyl)ethynyl (TIPSethynyl) groups as two independent steps. Gratifyingly, this revised strategy worked, leading to the first well-characterized and stable indeno[1,2-*b*]fluorene derivatives (14);^33^ however, this route suffered from three major pitfalls: (1) the transannular cyclization afforded no more than a hundred milligrams of tetraiodo material per run, and often much less; (2) the yields for preparing the octadehydrodibenzo[12]annulene were also low;^24^ and (3) the [12]annulene was prone to violent decomposition, as we (re)discovered.^34^

**Fig. 4:**
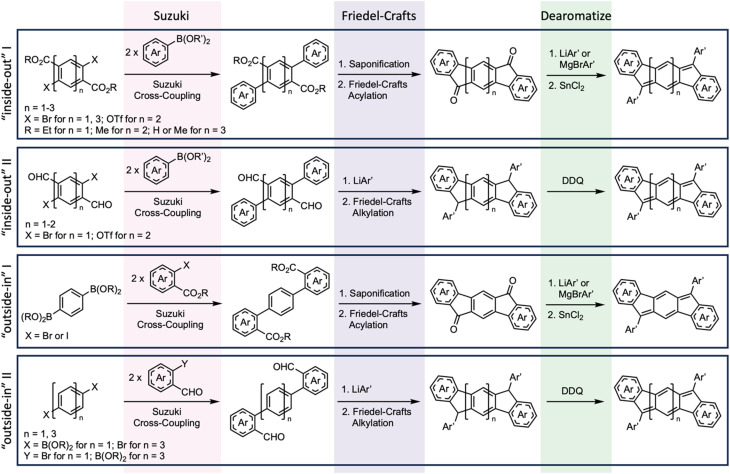
General synthetic strategy for the preparation of indenofluorenes and quinoidal analogues.

A new/improved synthetic route for IF synthesis was developed based on work of Deuschel^35^ and Wang.^36^ This modular and scalable strategy has been and continues to be our main method for preparing a wide variety of IFs and related quinoidal analogues from strongly diradical to strongly antiaromatic and spanning a variety of carbocycle- and heterocycle-fused rings (Fig. 4). The variations of the current syntheses, termed “inside-out” and “outside-in”, share three key steps: a Suzuki cross-coupling, Friedel–Crafts acylation or alkylation, and finally an oxidative or reductive dearomatization of the central arene. The “inside-out” route starts with the desired core, which possesses two halides (or pseudohalides) and either two esters or two aldehydes. The core is then Suzuki cross-coupled to outer arenes, which are typically functionalized with a boronic acid or boronate ester. If the resultant *para*-substituted core has two ester groups, saponification followed by Friedel–Crafts acylation yields the poorly soluble diketone precursor to the desired IF derivative (route I). Nucleophilic addition of bulky aryl or ethynyl groups, either by lithiate addition or Grignard addition, gives the penultimate diol precursor. Subsequent reductive dearomatization using SnCl_2_ yields the desired IF (Fig. 4, top entry).^37^ If the Suzuki cross-coupling furnished a *para*-substituted core with two aldehydes (route II), nucleophilic addition of the bulky aryl groups (by lithiate addition or Grignard addition) followed by Friedel–Crafts alkylation *via* the resultant secondary alcohol gives the dihydro precursor. Finally, oxidative dearomatization with DDQ yields the desired IF (Fig. 4, second entry).

The “outside-in” method follows the same steps as the “inside-out” but starts with the functional handles in reversed positions. This method only requires the core to have *para*-substituted boronic esters/acids while the outer arene cross-coupling partner bears the carbonyl functionality. After the Suzuki cross-coupling, the two “outside-in” routes parallel the “inside-out” methods—saponification, Friedel–Crafts acylation, nucleophilic addition of the aryl groups, and reductive dearomatization using SnCl_2_ (Fig. 4, third entry) or nucleophilic addition of bulky aryl groups, Friedel–Crafts alkylation, and oxidative dearomatization with DDQ (Fig. 4, last entry). While the “outside-in” route does lead predominantly to the formation of the [1,2-*b*]IF core, the Friedel–Crafts reaction can “close” the wrong way to produce a very small amount of the [2,1-*a*]IF isomer.^30*b*^ Given proper functionalization on the core and corresponding coupling partners, these two general methods provide ready access to a wide range of indenofluorene and related quinoidal topologies, allowing us to explore both core π-extension and outer arene modification.

**Fig. 5:**
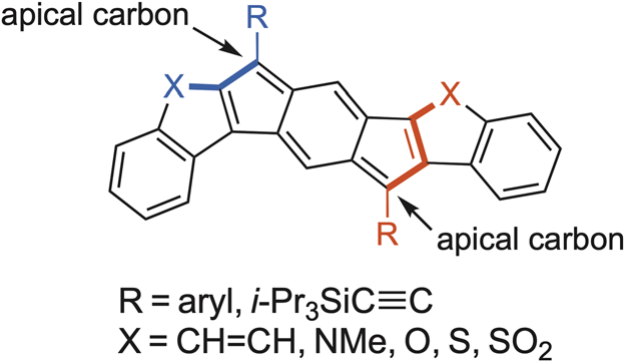
Generic carbocycle- or heterocycle-fused *s*-indacene with labelled apical carbons; *syn*-fusion is highlighted in blue and *anti*-fusion is highlighted in red.

As the new synthetic strategies were employed for increasingly complex systems, new naming practices became important to adopt. When fusing a simple benzene to *s*-indacene (as in the parent IFs), there are only two possible isomers (*e.g.*, [1,2-*b*]IF *vs.* [2,1-*b*]IF). Extending the parent [1,2-*b*]IF by one benzene on each side introduces three possible isomers, which were named *linear*-, *syn*-, and *anti*-, according to the direction of the angular naphthalene fusion.^38^ In the case of fusing heterocycles to an *s*-indacene core, there are two symmetric orientations possible. In one case, the heteroatom is on the same side as the apical carbon of the 5-membered core ring (*syn*-) and the other case the heteroatom is on the opposite side as the apical carbon (*anti*-) (Fig. 5).^39^ This naming convention is utilized throughout the remainder of this article.

### Initial studies

Admittedly, when we first started working on the 20 π-electron indenofluorenes, we assumed their high degree of conjugation might mean their electronic properties could be similar to the well-known 22 π-electron pentacenes,^40^ yet the IFs did not possess the Achilles' heel that leads to acene degradation, namely the reactive s*-cis* diene orientation of the double bonds. In many ways, our naïveté proved to be a blessing in disguise. Aside from this one (wrong) assumption of an indenofluorene/pentacene correlation, the slate was completely blank given the dearth of known indenofluorenes.

**Fig. 6:**
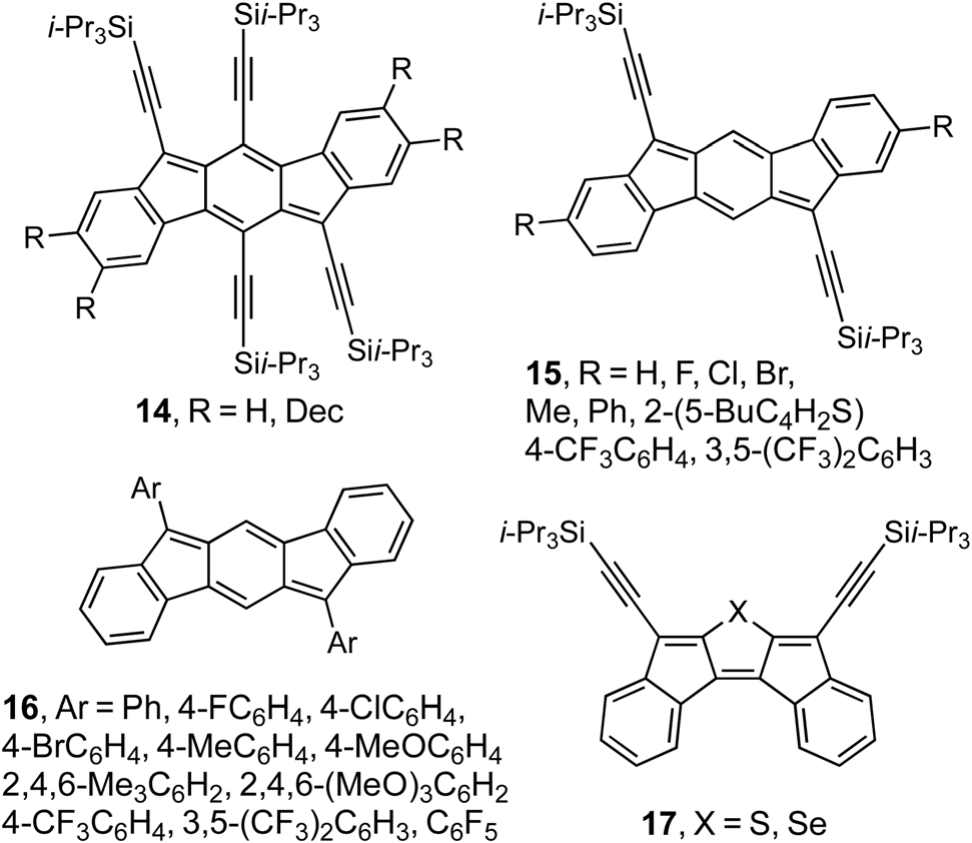
Early attempts to alter IF optoelectronic properties.

After our initial work on 14 showed that the [12]annulene transannular cyclization route was not scalable as well as potentially hazardous,^33,34^ graduate student Dan Chase switched to a variation of “inside-out” route I reported by Wang^36^ where the central piece was commercially available 2,5-dibromo-*p*-xylene. After the Suzuki cross-coupling, we oxidized the benzylic methyl groups with basic KMnO_4_ and then completed IF synthesis as outlined at the top of Fig. 4. While this route did provide a small library of stable donor- or acceptor-substituted [1,2-*b*]IFs 15 (Fig. 6),^41^ it too had drawbacks as some of the R substituents were not tolerant of the harsh KMnO_4_ oxidation. In addition, this study illustrated the need to examine the potential targets computationally before attempting their syntheses. Had we done so, we would have found that substitution on the 2- and 8-positions is undesirable as these carbons have minimal HOMO and LUMO orbital coefficients, relegating any influence to be through weaker inductive effects. Instead, the calculations suggested we should functionalize the 6- and 12-positions, which led to the series of [1,2-*b*]IFs 16.^42^ This proved much more successful, as we could vary the UV-Vis *λ*_max_ by ∼50 nm, whereas for 15 this variation of *λ*_max_ was a very modest 16 nm. Importantly, this latter study also demonstrated that we could prepare single-crystal OFETs with a [1,2-*b*]IF as the active layer that showed ambipolar transport, our first demonstration of the materials chemistry potential of the IFs,^42^ but more on that below.

One other area we examined briefly was to exchange the central benzene ring with either a thiophene or selenophene, leading to [2,1-*c*]IF analogues 17.^43^ While this structural variation altered the optoelectronic properties by ∼25 nm compared to the [2,1-*c*]IF, the *λ*_max_ were hypsochromically shifted much to our surprise. Furthermore, the difference between S and Se was minimal (1 nm), so further efforts on this pathway were abandoned. Rather than inclusion of the heterocycle within the antiaromatic core, we soon discovered that fusion of heterocycles to the core was the trick to generate highly antiaromatic structures (*vide infra*).

**Fig. 7:**
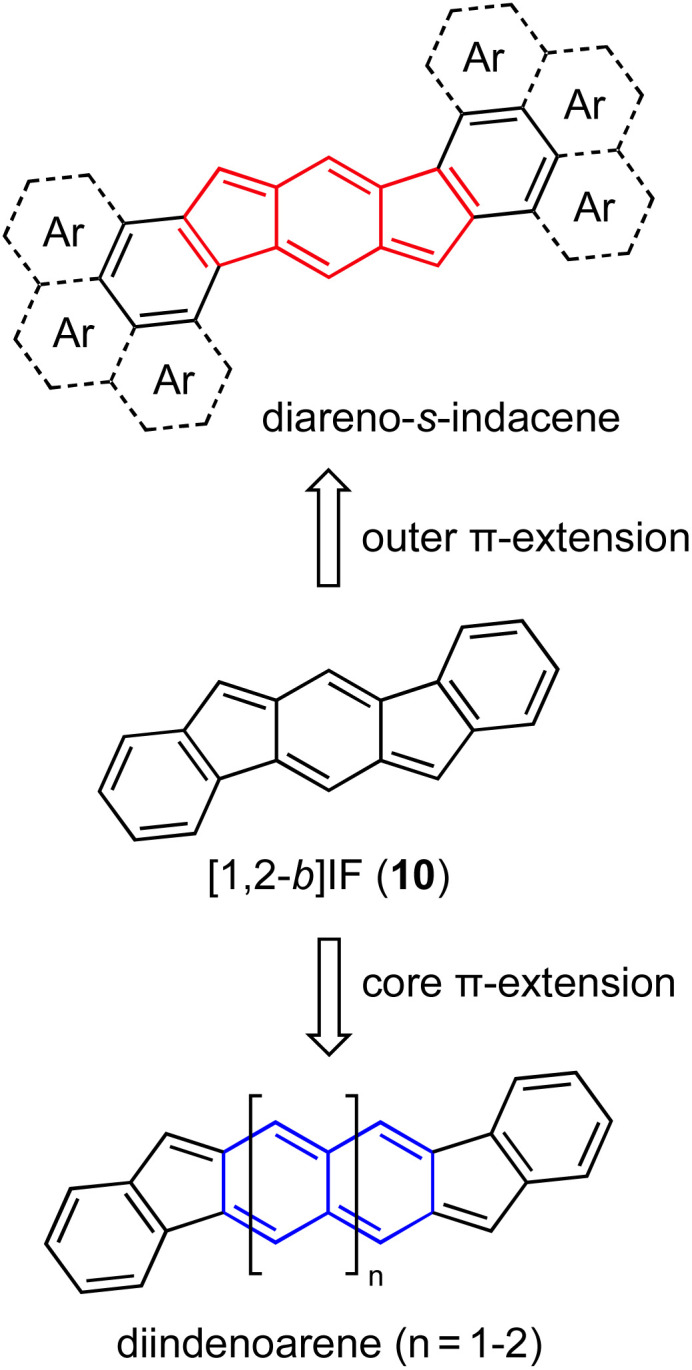
Modification of the [1,2-*b*]IF scaffold follows two main lines of investigation—outer π-extension/alteration with aromatic carbocycles or heterocycles (diareno-*s*-indacenes, top) and core π-extension from benzenoid to naphthoquinoid and anthraquinoid (diindenoarenes, bottom).

As noted earlier, the indenofluorenes sit at a unique intersection of antiaromaticity and diradical character. Modification of the indenofluorene scaffold has followed two main directions: core π-extension and outer π-extension (Fig. 7). Consistent with earlier computational studies on a series of dicyclopenta-fused acenes,^44^ we will show that expanding the quinoidal indenofluorene core by successive “benzinterposition”^17^ from one benzenoid ring ([1,2-*b*]IF 10) to two or three benzenoid rings (diindenoarenes), while significantly weakening core paratropicity, leads to strong diradical character in the latter systems.^45^ [1,2-*b*]IFs that possess an *s*-indacene core generally do not have strong diradical character; however, they do maintain a higher degree of antiaromaticity in the core. Such a transition from strong paratropicity to weak paratropicity and weak OS character to strong OS character is encouraged by the recovery of a Clar sextet in the OS resonance form stabilizing diradical character in the ground state in larger homologues.^13^ Concurrent exploration of the antiaromaticity and diradical character of these quinoidal systems has led to modification of the outer π-system to modulate the antiaromaticity within the *s*-indacene motif and thus translation of these findings about the outer arenes to π-expanded cores furnishes systems with tunable diradical character, thus highlighting the antiaromaticity/diradical character interrelationship.^13^ Modifications to rationally alter antiaromaticity will be presented first, followed by methods used to assess antiaromaticity and other inherent properties of these molecules. The use of different outer arenes to increase diradical character will be discussed next, and finally some device applications that take advantage of the antiaromaticity/diradical interrelationship will be described.

### Tuning the paratropicity strength of the *s*-indacene core by outer arene modification

While antiaromatic *s*-indacene derivatives have a potential role in the future of organic electronics, we first needed to better understand how to alter the strength of the antiaromatic *s*-indacene ring current in a rational manner, particularly through fusion of different arenes to the outside. Our first test of this hypothesis in 2014 fused thiophene (indacenodithiophene, IDT) and benzothiophene (indacenodibenzothiophene, IDBT) to 7.^39^ While the original idea was more curiosity driven and not motivated by increasing the antiaromaticity from the parent [1,2-*b*]IF, we found *via* Nucleus Independent Chemical Shift (NICS) calculations^46^ that these heterocycle-fused systems restored the paratropic ring current of the *s*-indacene motif close to that calculated for the parent hydrocarbon 7. This initially surprising result suggested that we needed to do a much deeper dive to elucidate the effects of outer arene alteration. Of course, this begs the question—what exactly are we “tuning”? The main effect is varying the HOMO/LUMO energy gap, with a secondary effect of altering the exact HOMO and LUMO energy levels (*vide infra*).

**Fig. 8:**
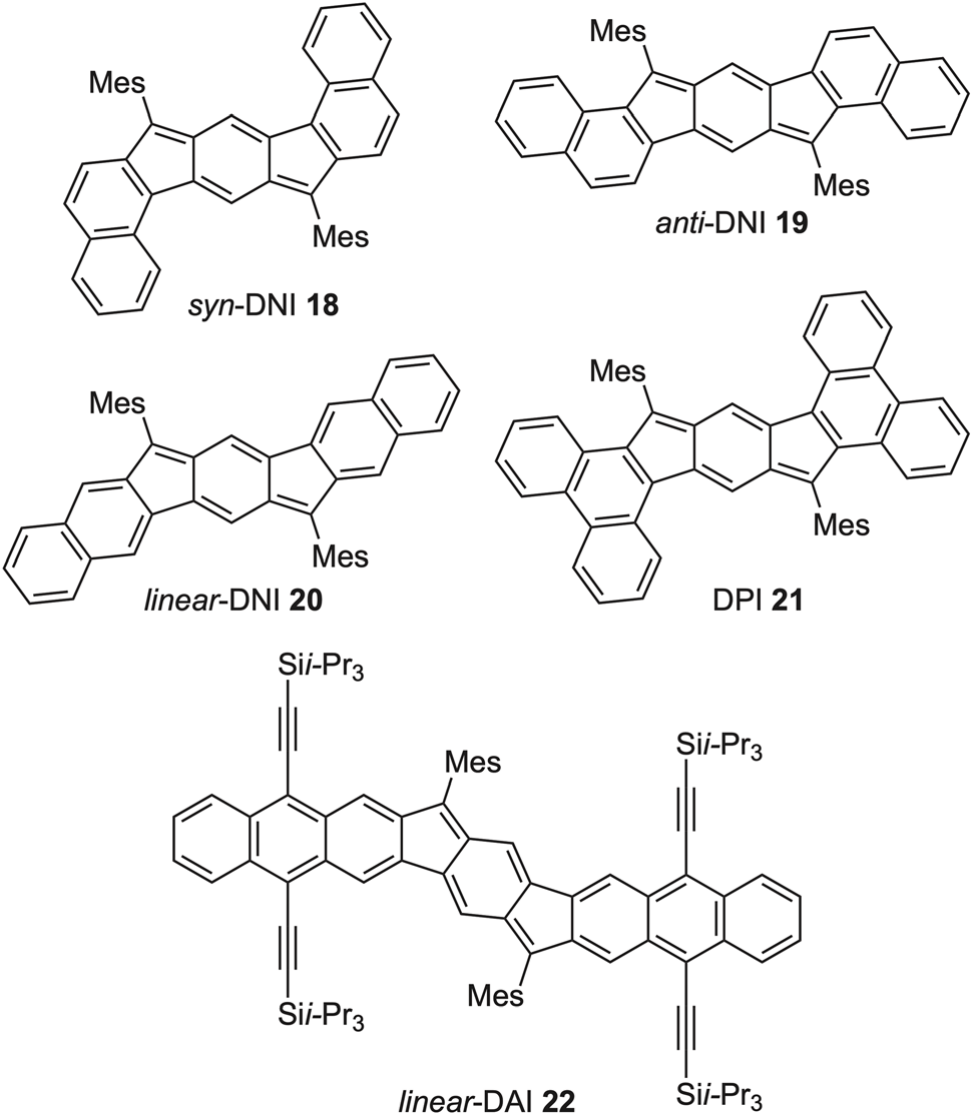
Aromatic carbocycle-fused *s*-indacene derivatives—DNIs 18–20, DPI 21, and DAI 22 (Mes = mesityl group).

Graduate student Conerd Frederickson initiated our first intentional foray into tuning the paratropicity of the *s*-indacene core by creating a series of molecules with fused aromatic carbocycles such as naphthalene (dinaphthoindacenes (DNI) 18–20, Fig. 8), phenanthrene (diphenanthroindacene (DPI) 21), and anthracene (dianthracenoindacene (DAI) 22).^38,47^ While 18, 19, and 21 showed core paratropicity values between those of [1,2-*b*]IF and the IDTs/IDBTs, both 20 and 22 indicated weaker core paratropicity than [1,2-*b*]IF. From these studies, a simple “bond order” rationalization^38^ suggested that greater double bond character of the fused bond (*e.g.*, the bond order of the fused bond in 21 is 1.8) resulted in increased antiaromaticity of the *s*-indacene core, whereas less double bond character of the fused bond (*e.g.*, bond order of fused bond in 22 is 1.2) resulted in decreased antiaromaticity. Interestingly, DAI 22 is the only fluorescent indacene derivative to date.^47^ The linear 2,3-anthracene fusion significantly diminished the paratropicity of the core of 22, resulting in an increase of the S_0_ → S_1_ energy gap and thus deactivating the low-barrier conical intersection observed in all other *s*-indacene derivatives.^48^ Nonetheless, this rudimentary “bond order” rationalization only works for the hydrocarbon-fused *s*-indacenes.^38^ In the realm of heterocycle-fused *s*-indacenes, the heteroatom plays an important role in affecting the antiaromaticity of the core, and this nuance is not captured simply by the bond order of the fused ring.

**Fig. 9:**
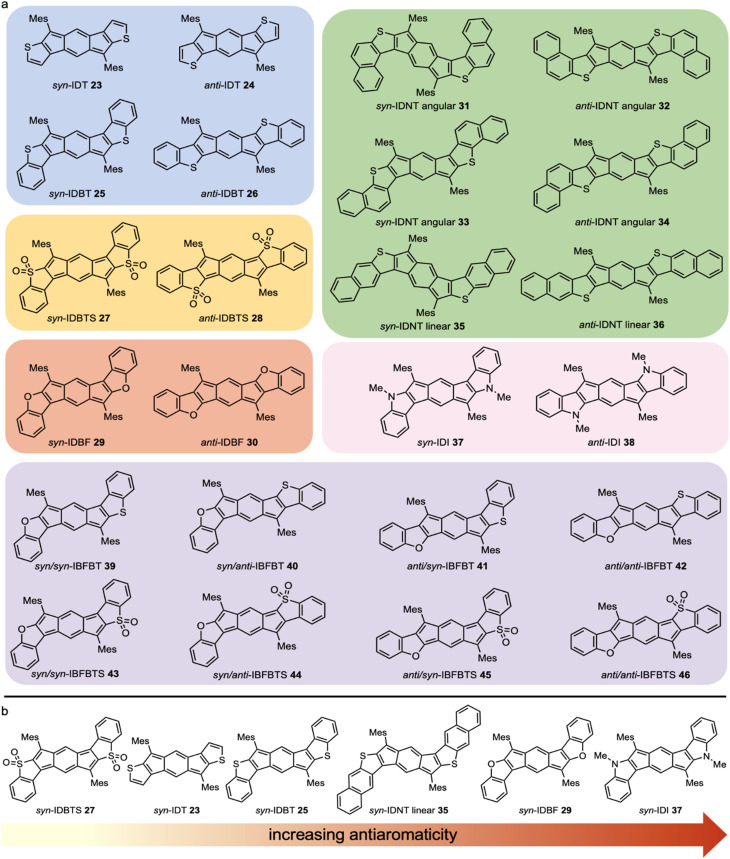
(a) Heterocycle-fused *s*-indacene core antiaromatics grouped by family of heterocycle: thiophenes/benzothiophenes 23–26 (blue), benzothiophene sulfones 27–28 (yellow), benzofurans 29–30 (orange), naphthothiophenes 31–36 (green), indoles 37–38 (pink), and mixed benzofurans/benzothiophenes 39–42 and their corresponding sulfones 43–46 (purple). (b) *syn*-Heterocycle-fused *s*-indacenes in order of increasing antiaromaticity.

After initially fusing thiophene (23–24, Fig. 9a) and benzothiophene (25–26) rings to *s*-indacene and observing an increase in antiaromaticity from the parent [1,2-*b*]IF,^39,49^ we became interested in further manipulating the antiaromaticity of the *s*-indacene core. Whereas fusion of arenes to 7 affects the degree of decreased paratropicity, fusion of heterocycles restores the antiaromaticity of 7 and has the potential to increase beyond the level of unsubstituted *s*-indacene itself. To further explore the effect of heterocycle fusion, three different directions were examined by grad students Justin Dressler, Gabby Warren, and Josh Barker, respectively: (1) oxidation of the benzothiophene-fused *s*-indacenes to sulfones (indacenodibenzothiophenesulfone (IDBTS) 27–28),^50^ (2) further π-extending the outer arenes from thiophenes and benzothiophenes to naphthothiophenes (indacenodinaphthothiophene (IDNT) 31–36),^51^ and (3) changing the heteroatom from sulfur to oxygen (indacenodibenzofuran (IDBF) 29–30)^52^ or nitrogen (indacenodiindole (IDI) 37–38).^53^ With the modular synthetic routes available (Fig. 4), if the desired outer arene cross-coupling partner could be accessed, the “inside-out” method yielded the desired isomers. The ultimate examples of this precision synthetic method were showcased in the preparation of unsymmetrical, regioisomeric indacenobenzofuranbenzothiophenes (IBFBTs) 39–42 and their corresponding oxidized donor/acceptor IBFBTS counterparts 43–46*via* selective Suzuki cross-coupling to dimethyl 2-chloro-5-iodoterephthalate,^54^ whereas such specific, highly defined molecular geometries in acenes and related structures would be difficult to produce given current preparative methods (*e.g.*, regio-random Aldol condensation).^40^ While a majority of these *s*-indacenes were stable and could be thoroughly characterized, a few either were short-lived (30, 46) or afforded something other than the desired product (38).

### Computational techniques

**Fig. 10:**
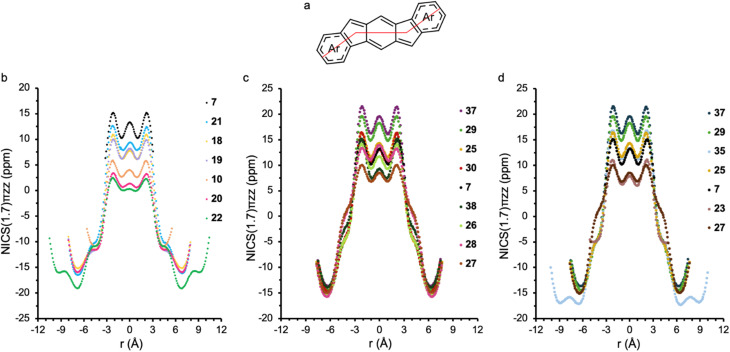
(a) Example path of the dummy atoms in NICS-XY(1.7)_πZZ_ scan calculations. NICS-XY scans of (b) carbocycle-fused systems (10 and 18–22) and (c) heterocycle-fused systems (25–30 and 37–38) *versus* that of *s*-indacene reference molecule 7. (d) NICS-XY scans of only the *syn*-fused heterocycles in Fig. 9b. All NICS scan plots are ordered from most to least antiaromatic. NICS-XY scans performed at 1.7 Å above the ring system using geometries optimized at the M11/6-311 + G** level of theory.

When evaluating the antiaromaticity of *s*-indacene derivatives, we employ a variety of experimental and computational techniques. Our most powerful and useful computational tool is NICS-XY scans.^55^ This convenient and information-rich method plots the NICS values of dummy atoms placed in a set path across the compound of interest (Fig. 10a), making it visually simple to identify trends in aromaticity and antiaromaticity across a series of molecules (Fig. 10b–d). Using NICS-XY scans, students in the Haley lab identified several new substituted *s*-indacene targets with pronounced paratropicity (*e.g.*, 29–38), as they were predicted to be equal to or even more antiaromatic than our benchmark, unsubstituted *C*_2h_*s*-indacene 7.^51–53^

Fig. 10 includes NICS-XY(1.7)_πZZ_ scans (*σ* only model) for selected carbocycle- (10 and 18–22) and heterocycle-fused compounds (25–30 and 37–38) with benchmark 7. It should be noted that these are recently recalculated/newly published NICS-XY scans based on M11-optimized geometries,^56^ as Wu and co-workers showed that NICS-XY scans based on B3LYP-optimized geometries, as we originally published, can often provide values that overestimate delocalization in the *s*-indacene core.^57^ It is also important to note that one should not focus solely on the absolute NICS values but rather on the trends in data that are all calculated with the same parameters. For the carbocycle series (Fig. 10b), *s*-indacene 7 is the most antiaromatic followed by phenanthro-fused 21, the *syn*/*anti*-DNI isomers (18/19), the parent [1,2-*b*]IF (10), and then the *linear*-analogues (20/22), supporting the simple “bond order” rationalization for paratropicity strength.

Whereas the differences between *syn*- and *anti*-fusion are minimal in the carbocycles, the differences are much more pronounced in the heterocycle series (Fig. 10c). In general, *syn*-fused aromatic heterocycle isomers are more antiaromatic than the *anti*-fused isomers, with the *syn-*indole (37), -benzofuran (29) and -benzothiophene (25) all more paratropic than benchmark 7 and *anti*-indole (38), -benzofuran (30) and -benzothiophene (26) possessing roughly comparable paratropicity to 7. Interestingly, the nonaromatic benzothiophene sulfone-fused derivatives 27 and 28 are calculated to be less antiaromatic than 7 and are the only heterocycle-fused *s*-indacenes that reverse the trend of increased antiaromaticity for *syn*-fusion, *i.e.*, *anti*-IDBTS 28 is more paratropic than *syn*-IDBTS 27.

While Fig. 10b–d show an appreciable ∼20 ppm variance in the NICS_πZZ_ values of the *s*-indacene core, the corresponding values of the external rings are essentially insensitive to bond fusion/bond order. In the carbocyclic series, the range of NICS_πZZ_ values for the ring directly fused to *s*-indacene is small (−10 to −12 ppm), and even smaller (−15 to −16 ppm) for the terminal ring of the DNIs. Whereas the values of the inner ring show the ‘conflict’ between opposing diatropic and paratropic ring currents, the outer ring is more isolated and thus the values are more typical of benzene/naphthalene. Exchanging the fused carbocyclic ring for a heterocyclic ring significantly negates this ‘conflict’, as the now-weakly diatropic heterocycles (NICS_πZZ_ values of −2 to −4 ppm) or atropic sulfone 27 (+1 ppm) act as a spacer separating the strongly diatropic outer benzenes and the highly paratropic *s*-indacene.

While our studies had shown that extending the π-system of *s*-indacene through heterocycle or benzoheterocycle annelation leads to modification of the antiaromatic character of the core, we did not know whether this was a result of extension of the π-system or the presence/location of the heteroatom. To understand these differences, we examined a series of test molecules to elucidate the specific effects of the heteroatom and its substitution position, the heterocycle, and the further extension of π-conjugation.^58^ Computationally this was accomplished using NICS calculations,^46^ current density maps,^59^ and NICS2BC,^60^ all of which painted a consistent picture. First, we found that the heteroatom itself can have a dramatic effect on the antiaromaticity of the core: placing uncyclized heteroatoms at the 1,5-positions (*i.e.*, the *anti*-isomers) affords the greatest extent of antiaromaticity alleviation, leading in the case of the NH_2_-substituted *s*-indacene to almost complete loss of antiaromatic character. Second, we established that this effect is highly site-specific. Using resonance structures, we rationalized this site-sensitivity by showing that the *anti*-position enables the substituent to interact with the core *via* resonance while the *syn*-position does not. Third, by comparing the two types of isomers we found that the extension of the π-system by heterocycle annelation does indeed alleviate the antiaromaticity of the core, although it is a weaker effect than the resonative heteroatom effect. Finally, we demonstrated that this effect is dampened by the addition of another ring, *i.e.*, benzannelation. Overall, this study showed that the reduction in antiaromaticity in the *anti*-isomers is primarily a heteroatom effect, which is weakened by cyclization and further benzannelation. In contrast, for the *syn*-isomers (Fig. 9b) the heteroatom effect is weak and the majority of antiaromaticity alleviation is achieved by the neighboring ring effect; however, further benzannelation cancels this, allowing the *s*-indacene to regain (and even surpass) its full antiaromaticity, as illustrated in Fig. 10d.^58*a*^

### NMR spectroscopy

^1^H NMR spectroscopy is a routine part of characterizing new *s*-indacene derivatives and provides an additional way to compare the paratropicity of similar systems. The protons on the six-membered ring of the *s*-indacene core are shifted upfield relative to the degree of paratropicity—the more antiaromatic molecules show a greater upfield shift of the core protons. When the bulky aryl groups are consistent, *e.g.*, the mesityls in Fig. 8 and 9a, comparisons between different systems become possible. This is a powerful tool for several reasons: NMR spectroscopy is a magnetic-based method, making it easy to compare with magnetic criteria based computational methods such as NICS, and it can be compared across a wider range of systems than cyclic voltammetry (CV) or UV-Vis spectroscopy, for example.

In most cases, comparison of the chemical shift of the core protons matches the order predicted by the NICS-XY scans. Table 1 shows the collected values for *s*-indacene core IF analogues. NICS-XY scans predict *syn*-fusion of the sulfur heteroatom to increase antiaromaticity through π-extension (IDT 23 → IDBT 25 → IDNT 35), and this matches with the further upfield chemical shift of the core proton: IDT 23 (6.06 ppm) > IDBT 25 (6.02 ppm) > IDNT 35 (5.99 ppm). While the ordering within the six IDNT regioisomers does not follow the NICS-XY scans perfectly, the differences are small and other effects sometimes dominate.^51*b*^ The oxidized IDBTSs reverse in trend and the *anti*-fusion has the more upfield chemical shift (28, 6.03 ppm) than the *syn*-fusion (27, 6.91 ppm).^50^ Through various modifications to the sulfur-based heterocycle-fused IFs, we notice a significant upfield shift from the parent IF and smaller changes within the family. Consistent with the computational studies,^58*a*^ changing the heteroatom from sulfur to oxygen or nitrogen leads to a dramatic upfield shift of the core proton to 5.60 ppm for *syn*-IDBF 29^52^ and 5.39 ppm for *syn*-IDI 37.^53^ From these trends we can conclude that large changes to antiaromaticity can be made through heteroatom alteration, and fine tuning antiaromaticity is done through fusion orientation (*syn*-/*anti*-), π-extension (IDT, IDBT, IDNT), and electronics (thiophene *vs.* thiophene sulfone). Finally, the core proton shifts of the unsymmetric IBFBT systems can be thought of as an “average” of their two constituent pieces, *e.g.*, the core protons of *syn*,*syn*-IBFBT (5.90, 5.80 ppm) are roughly the average of those in *syn*-IDBT 25 (6.02 ppm) and *syn*-IDBF 29 (5.60 ppm).^54^

**Table 1:** ^1^H NMR chemical shift of the core protons of select *s*-indacene derivatives

Cmpd	*δ* (ppm), (solvent)	Cmpd	*δ* (ppm), (solvent)
7 (Ar = Mes)^a^	6.51 (CDCl_3_)	16 (Ar = Mes)	6.86 (CDCl_3_)
18	6.68 (CD_2_Cl_2_)	19	6.62 (CD_2_Cl_2_)
20	7.14 (CDCl_3_)	21	6.95 (CDCl_3_)
22	7.19 (CDCl_3_)	—	—
23	6.06 (CDCl_3_)	24	6.05 (CDCl_3_)
25	6.02 (CDCl_3_)	26	6.07 (CDCl_3_)
27	6.91 (CDCl_3_)	28	6.03 (CDCl_3_)
29	5.60 (CD_2_Cl_2_)	30	6.14 (CD_2_Cl_2_)
35	5.99 (CDCl_3_)	36	6.09 (CDCl_3_)
37	5.39 (CD_2_Cl_2_)	—	—
39	5.85 (CD_2_Cl_2_)^b^	40	5.87 (CD_2_Cl_2_)^b^
41	6.05 (CD_2_Cl_2_)^b^	42	6.10 (CD_2_Cl_2_)^b^
43	6.17 (CD_2_Cl_2_)^b^	44	5.64 (CD_2_Cl_2_)
45	6.50 (CD_2_Cl_2_)^b^	46	5.99 (CD_2_Cl_2_)

a Ref. 22.

b Average of two peaks.

### Electrochemical and photophysical properties

The electrochemical and photophysical properties of the diarenoindacenes are also indicative of their antiaromaticity. While they possess HOMO–LUMO energy gaps and thus low energy absorptions similar to acenes, the diarenoindacenes are characterized by considerably lower HOMO and LUMO energy levels, typical of antiaromatic molecules. Additionally, these systems are redox active and usually have two reduction and one or two oxidation events. Unlike NMR spectroscopy, it is more difficult to draw meaningful comparisons of the photophysical and electrochemical properties across a wide range of derivatives. The HOMO–LUMO gap is affected by both antiaromaticity and π-extension, making it difficult to deconvolute their combined impact. Similarly, direct comparison of photophysical properties across families of diarenoindacenes is complicated by the same factors, resulting in red-shifted absorptions; however, both CV and UV-Vis provide important information about the system and its antiaromaticity, especially the ability to tune the HOMO–LUMO energy gap.

Table 2 contains the HOMO and LUMO energy levels, HOMO–LUMO energy gaps, and *λ*_max_ of the low energy absorption of a variety of diarenoindacenes. In general, we see that the more antiaromatic systems (25, 29, and 35) have a smaller HOMO–LUMO energy gap and more red-shifted absorption, especially when comparing the *syn*- and *anti*-isomers of a particular heterocycle. The broad class of diarenoindacenes typically has HOMO and LUMO energy levels in the range of −5.5 to −5.8 and −3.7 to −4.1 eV, respectively, with HOMO–LUMO energy gaps in the range of 1.4–2.0 eV. Interestingly, oxidation of the benzothiophenes to their corresponding sulfones (27/28/43) further drops the LUMO energy level (−4.4 to −4.5 eV). The low LUMO energy levels reflect the high electron affinity of the diarenoindacene family, which stand in contrast to acenes, which typically have much lower electron affinities unless heavily appended with electron-withdrawing groups. These molecules' pronounced electron affinities make intuitive sense—as they are successively reduced, the compounds regain an aromatic configuration and thus aromatic stabilization. Much like the fullerenes, the presence of the five-membered rings containing all-sp^2^ hybridized carbons makes these compounds inherently electron-accepting.

**Table 2:** Electrochemical and photophysical values of select *s*-indacene derivatives

Cmpd	*E*_HOMO_ (eV)	*E*_LUMO_ (eV)	*E*_gap_ (eV)	*λ*_max_ (nm)
16 (Ar = Mes)	−5.34	−2.92	2.42	516
18	−5.73	−3.72	2.01	578
19	−5.59	−3.68	1.91	549
20	—	—	—	543
21	−5.73	−3.87	1.86	622
22	—	—	—	615 (em 664)
25	−5.54	−3.93	1.61	626
26	−5.52	−3.81	1.71	618
27	−6.28	−4.51	1.77	624
28	−6.12	−4.46	1.66	587
29	−5.55	−3.97	1.58	642
30	—	—	—	584
35	−5.49	−4.08	1.41	697
36	−5.43	−3.83	1.60	665
39	−5.67	−4.08	1.59	630
43	−5.84	−4.44	1.40	670

The diarenoindacenes are highly colored, covering a broad spectrum with colors varying from orange (23) to red-orange (16a) to purple (27) to blue (36), which are reflected in the electronic absorption spectra of the molecules. Compared to 16 (516 nm, Table 2), the *λ*_max_ of the low energy band of the carbocycle-fused indacenes progressively shifts to the red—543 (20), 549 (19), 578 (18), and 622 nm (21), decreasing the optical gap from *ca.* 2.3 to 1.9 eV.^38^ Although not shown in Table 2, replacement of the mesityls with (triisopropylsilyl)ethynyl (TIPSethynyl) groups typically shifts the low-energy *λ*_max_ further into the red by roughly 50–75 nm (*e.g.*, low-energy *λ*_max_ for TIPSethynyl-substituted 16 and 21 are 568 and 692 nm, respectively). This difference is attributable to the degree of the conjugation of the quinoidal core with apical substituents: whereas the alkynes are fully conjugated to the π-electron-rich cores, the mesityls are only in partial π-electronic communication because of their near orthogonality (>75° dihedral) to the diarenoindacene backbone.

Despite being highly colored and π-electron-rich systems, the diarenoindacenes are surprisingly non-fluorescent due to a non-radiative decay pathway,^48^ as was also observed in earlier studies of *s*-indacene derivatives.^61^ Transient absorption spectroscopy of derivatives of 10 and 13 revealed that the molecules possess extremely short S_1_ lifetimes of ∼10–15 ps.^48^ Interestingly, studies on the tetrakis(*t*-butyl) analogue of core structure 7 showed it to have a lifetime of 18 ps for the S_1_ to ground state relaxation.^61*a*^ Such extremely short lifetimes explain why these molecules are non-emissive, since fluorescence (typically occurring with lifetimes of greater than 10^−9^ s) is not a competitive process at this time scale. Quantum chemical calculations indicate this non-emissiveness to be the result of an easily accessible potential energy surface crossing between the first excited singlet state (S_1_) and ground electronic state (S_0_), *i.e.*, a conical intersection.^48,61^ Similar to 7, this process allows efficient internal conversion to the ground state, deactivating fluorescence and thus yielding ‘dark’ molecules. As noted earlier, the only exception to this case is DAI 22—with its antiaromaticity turned ‘off’ by 2,3-anthraceno-fusion, the S_0_ and S_1_ states in 22 sufficiently separate in energy that fluorescence, albeit weak, is restored.^47^

### X-ray crystallography

X-ray crystallography and its use in determining bond lengths has been an important factor in understanding our *s*-indacene derivatives. We find that when fusing arenes or heteroarenes to an *s*-indacene core, we observe a bond localized structure similar to *C*_2h_ symmetric *s*-indacene 7. Applying X-ray crystallography to understand the change in antiaromaticity in the IF scaffold focuses on the bond length alternation in the *s*-indacene core. Generally, we observe that the structures with a greater degree of antiaromaticity have more bond length alternation, *e.g.*, the short bonds are shorter and the long bonds are longer as antiaromaticity increases. While this metric provides support for ranking the antiaromaticity of IF derivatives, it also has some pitfalls. In addition to the complex relationship between antiaromaticity and bond lengths, crystal packing forces are known to affect bond lengths and make bond length analysis difficult,^22^ and some antiaromatic compounds can also be delocalized.^62^
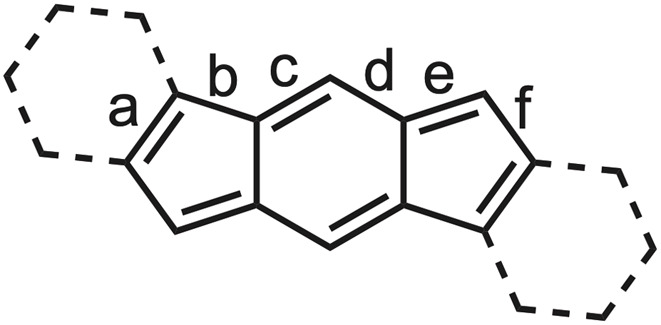


**Fig. 11:**
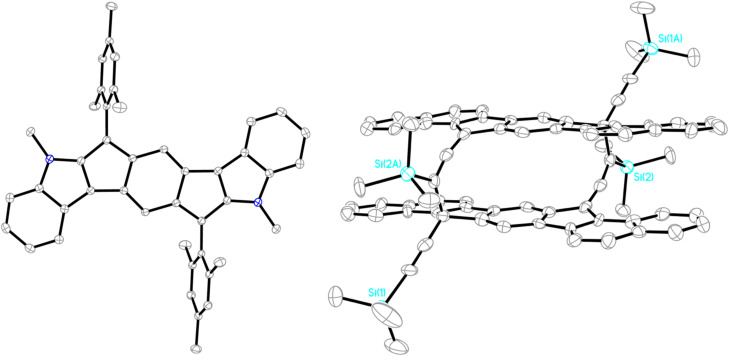
Molecular structures of (left) *syn*-IDI 37 (CCDC 2416146) and (right) the dimer of the bis(trimethylsilyl)ethynyl-substituted analogue of 19 (CCDC 2416145). Ellipsoids are drawn at the 50% probability level; hydrogen atoms omitted for clarity.

Table 3 lists the core bond lengths of mesityl-substituted 16, 18, 22–30, and 35–37. All the structures exhibit the *para-*quinoidal motif of closed shell 6 within the indacene core, with bonds c and e possessing more double-bond character clearly evident, *e.g.*, *syn*-IDI 37 in Fig. 11 (left). For example, double bond c in the central six-membered ring varies from 1.356 to 1.386 Å. Bond e between the core and the apical carbon bearing the mesityl groups varies from 1.371 to 1.380 Å in the hydrocarbon structures, yet lengthens to 1.389–1.435 Å in the heterocycle-fused systems, which begins to hint at the contribution of a diradical resonance form such as open-shell 6. The outer benzenes exhibit archetypical bond lengths of 1.38–1.40 Å, with the fused bond at ∼1.41 Å. A large majority of the diarenoindacene structures are well-bracketed by these values, whether aryl- or alkynyl-substituted on the apical carbons. In addition, nearly all the molecules are planar species, with root-mean-square deviations from the average molecular plane of 0.04 Å or less. Interestingly, sulfone-fused *s*-indacenes 27 and 28 exhibit a “flipped” bond alternation within the indacene core, in which the double bonds are exocyclic to the heterocycle.^50^ This flipping relieves the potential destabilizing effects of enhanced paratropicity if the bond alternation were to retain a normal pattern.

**Table 3:** Core C–C bond lengths (Å) of select *s*-indacene derivatives

Cmpd	a	b	c	d	e	f
16 (Ar = Mes)	1.413	1.469	1.356	1.433	1.380	1.471
18	1.401	1.470	1.367	1.431	1.377	1.461
22^a^	1.450	1.447	1.368	1.418	1.378	1.452
23	1.384	1.461	1.363	1.418	1.398	1.447
24	1.389	1.452	1.360	1.431	1.388	1.460
25	1.391	1.457	1.371	1.421	1.407	1.441
26	1.393	1.437	1.377	1.412	1.409	1.435
27^a^	1.442	1.397	1.415	1.371	1.467	1.376
28	1.437	1.384	1.420	1.370	1.464	1.375
29	1.374	1.451	1.371	1.418	1.418	1.432
30	1.390	1.412	1.386	1.394	1.435	1.419
35	1.373	1.468	1.368	1.422	1.389	1.438
36^a^	1.397	1.433	1.376	1.406	1.413	1.429
37^a^	1.382	1.458	1.363	1.429	1.394	1.448

a Average of two independent molecules in the crystal lattice.

As noted earlier, all five IF isomers possess some degree of diradical character (Fig. 3b). Over the course of our [1,2-*b*]IF studies, we rarely saw evidence of this reactivity, likely because of our use of bulky mesityl and/or (triisopropylsilyl)ethynyl groups on the apical carbons. Interestingly, Fu and Zhao found in 2015 that the bis(trimethylsilyl)ethynyl analogue of 15 can afford dimeric, trimeric, and oligomeric materials.^63^ An X-ray structure of the dimer showed that the radical center could delocalize onto the carbon next to the silicon atom, yielding a cyclophane dimer with four allene linkages. We tried this same strategy on the bis(trimethylsilyl)ethynyl-substituted analogue of 19 and were surprised by the resulting structure (Fig. 11, right).^64^ This too afforded a dimeric cyclophane, but only two of the four alkynes had “isomerized” to the allene motif. Rather than an allene–allene linked dimer, one allene was linked to the apical carbon of its neighboring indacene core. Clearly such a structure must arise from the latent diradical character of the [1,2-*b*]IF scaffold. During the revisions stage of this manuscript, Tobe disclosed the analogous dimerization process in 10,12-bis(trialkylsilyl)ethynyl derivatives of [2,1-*b*]IF 12, which they attributed to attractive London dispersion forces operating between the π-system and the alkyl groups.^65^

### Dicyclopenta[*b*,*i*]anthracene: the core to achieve strong OS character

**Fig. 12:**
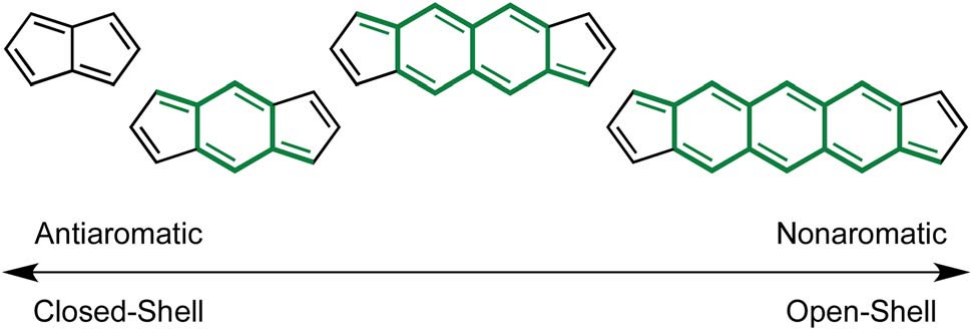
Continuum of core PAAHs from antiaromatic to diradicaloid featuring sequential insertion of 6-membered rings, *i.e.*, benzinterposition. Pro-aromatic *p*-QDM, 2,6-naphthoquinodimethane (NQD) and 2,6-anthroquinodimethane (AQD) fragments are highlighted.

After establishing a large body of work on indenofluorenes with multiple strategies for external acene and heterocycle fusion on *s*-indacene to tune optoelectronic properties and paratropicity, the next logical progression was internal π-expansion featuring dicyclopenta[*b*,*g*]naphthalene (DCN) and dicyclopenta[*b*,*i*]anthracene (DCA) cores, *e.g.* sequential benzinterposition, to investigate further changes in optoelectronic properties. However, the relationship between antiaromaticity and diradical character discussed above sees a greater shift towards OS character proportional to molecule size and can be thought of as a continuum from antiaromaticity to diradical character (Fig. 12).^13,44*c*,66^ While unintended at the time, the Haley lab would find itself entering the arena of stable diradicaloids on account of the elongated quinoidal conjugation of proaromatic fragments within the DCN and DCA cores. Admittedly, core expansion did begin with fluoreno[4,3-*c*]fluorene ([4,3-*c*]FF),^67^ but discussion of the FF homologues will be presented later as they uniquely straddle CS and OS behavior. The focus here will instead be on DCA-based diindeno[1,2 *b*/1′,2′-*i*]anthracene (DIAn) homologues as we now know they are strong diradicaloids and serve as the upper limit to OS character studied in our lab so far.

**Fig. 13:**
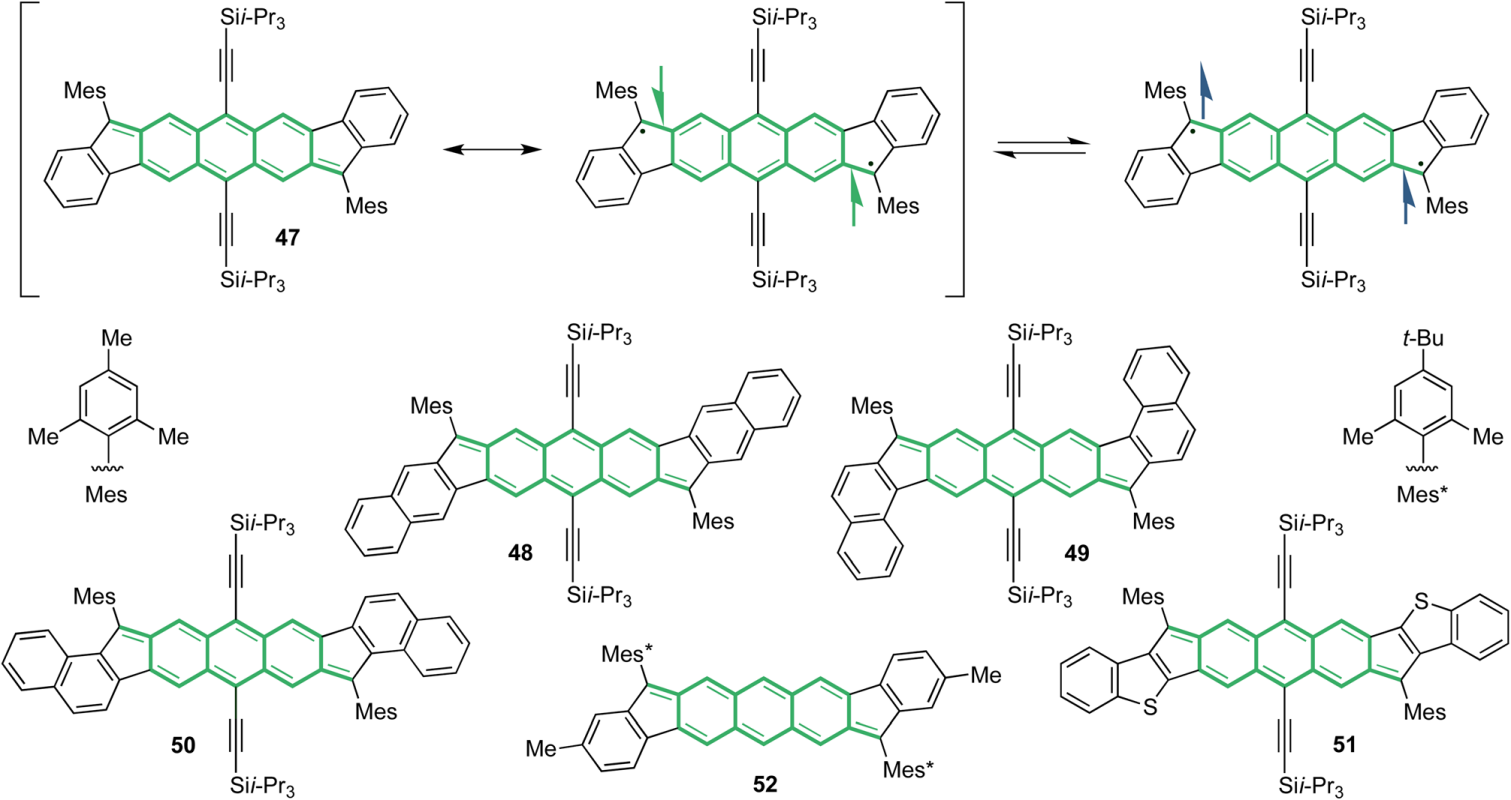
CS and OS resonance structures of DIAn 47 depicting transition from singlet to triplet states alongside DIAn homologues 48–52. Pro-aromatic AQD fragments are highlighted in green.

Pursuit of DIAn 47 (Fig. 13) had begun in 2012 motivated by anticipation of enhanced optoelectronic properties, but the project remained slow going until then graduate student, Gabriel Rudebusch, completed the synthesis in 2015 following our “outside-in” I strategy.^68^ To his credit he rationalized that inclusion of both the bulky mesityl and TIPSethynyl groups in the starting material would impede the Friedel–Crafts alkylation at the favored 1/5-positions of the anthracene core because of steric clash, but rather close onto the less favored 3/7-positions, which worked brilliantly. Treatment with DDQ afforded deep blue crystals of 47. Interestingly, the peaks in the proton NMR spectrum at room temperature were slightly broadened, so Rudebusch performed variable-temperature ^1^H NMR (VT-NMR) experiments up to 150 °C to qualitatively probe a singlet to triplet state transition. Upon warming, the aromatic peaks in the series of spectra gradually broadened as the paramagnetic triplet state was populated. By 150 °C, all the peaks were in the baseline save those of the nondeuterated NMR solvent, and all the peaks returned to their former shape and height upon cooling to room temperature, offering the first evidence of having a genuine diradicaloid. In the electronic absorption of 47, a major low energy absorbance at 690 nm was accompanied by a weak shoulder starting near 725 nm and tailing to 900 nm. This peculiar profile hints towards OS character as shoulders extending into the near-infrared (NIR) region have been attributed to the doubly-excited configuration in diradicaloids.^69^ The system also exhibited textbook redox amphoterism *via* cyclic voltammetry as two quasi-reversible single-electron oxidations and reductions were recorded, affording a particularly narrow *E*_gap_ of 1.45 eV, which is a much smaller value compared to its relative [1,2-*b*]IF cousins (2.22 eV). A single crystal of DIAn suitable for X-ray diffraction was then grown and analyzed. Of particular importance was the 1.406 Å bond length between the apical carbon and the anthracene core, suggesting this bond was displaying some single bond character reflecting the OS resonance structure. Other elongated double bond lengths in the crystal structure suggested there was substantial contribution from the OS form in the ground state canonical structure (Fig. 13). These findings frame DIAn as a stable singlet diradicaloid, especially when paired with its *y*_0_ value of 0.623. It is worth noting that, while it is possible to delocalize the radical center onto the arene core of 47 (and the related other diradicaloids described below), the odd-electron density maps consistently show that the apical carbon is the site of highest spin density.^68,70^

### Altering singlet-triplet energy gaps and electronic parameters

Inspired by our work with IFs, naphthalene fusion to the periphery of the DIAn scaffold affords three dibenzodiindenoanthracene (DBDIAns) isomers 48–50 that mirror the *linear*-, *syn*- and *anti*-fusions seen in IFs 18–20. With such π-extension, the diradicaloid character sequentially strengthens from 48–50 covering a *y*_0_ range of 0.623–0.711 for the hydrocarbon DIAns (Table 4).^70^ Benzothiophene fusion in 51 pushes OS character even further (*y*_0_ = 0.812) and effectively caps our suite of diradicaloids as the upper bound. Each analogue exhibited prominent diradicaloid signatures in VT ^1^H NMR and electronic absorption experiments with spin-state transitions qualitatively observed in NMR signal broadening along with double-excitonic features in the NIR region. Elongated double bonds between the apical carbon and anthracene core in the crystal structures (1.391–1.434 Å) also supported substantial OS character.

**Table 4:** Electronic parameters and singlet–triplet energy gap for DIAn derivatives 47–52

Cmpd	*U*/2 (eV)^a^^,^^b^	*t*_ab_ (eV)^a^^,^^b^	*y*_0_ a ^,^ c	Δ*E*_STcalc_ (kcal mol^−1^)^a^^,^^d^	Δ*E*_STexpt_ (kcal mol^−1^)
DIAn 47	1.435	0.916	0.623	−4.71	−4.2
linear-DBDIAn 48	1.348	0.905	0.638	−5.09	−4.8
*syn*-DBDIAn 49	1.378	0.818	0.686	−4.16	−3.8
*anti*-DBDIAn 50	1.377	0.781	0.711	−3.45	−3.2
DBTDIAn 51	1.572	0.774	0.815	−3.41	−3.4
DIAn 52	1.463	0.957	0.615	−5.42	−4.6

a Geometries optimized at R- and UB3LYP/6-311G* levels.

b Estimated at CASCI(2,2)6-311G* level using (tuned-)LC-RBLYP MOs (denoted as tuned-LC-RBLYP-CASCI(2,2)/6-311G*).

c Calculated at the PUHF/6-311G* level.

d Adiabatic Δ*E*_ST_ value calculated at the spin-flip noncollinear (SF-NC)-TDDFT PBE5050/6-311G* level along with R- or UB3LYP/6-311G* zero-point vibrational energy correction for each spin state.

Whereas the initial synthetic design of DIAn 47 included TIPSethynyl groups to direct the correct closure during the Friedel–Crafts alkylation step, further synthetic refinement allowed us to switch to an ‘inside-out’ ring-closing strategy, resulting in DIAn 52 with no TIPSethynyl groups attached to the DCA backbone. We suspected that the ethynyl groups were electronically non-innocent because of a *y*_0_ value shifting to 0.615.^70^ Investigation of 52 revealed a very similar UV/Vis profile and X-ray structure to 47, so optoelectronic properties were minimally impacted by loss of alkyne substitution.^71^ Even so, the lower *y*_0_ suggests there is a change to the diradicaloid character. While the computational and experimental evidence so far begins to paint a reasonably complete image of our diradicaloids, the finer details regarding changes in spin-state transitions between DIAn derivatives were not entirely clear. To probe deeper into the OS nature of our DIAns, it was necessary for our collaborators in the Nakano group at Osaka University to dig computationally into the details.

The energy gap between the singlet state and triplet state (Δ*E*_ST_) in diradicaloids is an important property that can be calculated and then unambiguously measured experimentally to quantify diradical character. Calculations using the two-electron/two-site model were carried out to probe the parameters contributing to Δ*E*_ST_. Eqn (1) and (2) describe the model's determination of Δ*E*_ST_ and its relationship to *y*, where *a* and *b* represent the two electrons in localized natural orbitals, with several key terms: *U* is defined as the difference between onsite and intersite coulomb repulsions, *t*_ab_ is the transfer integral, and *K*_ab_ as the direct exchange integral. Within this framework, the balance between *U* and *t*_ab_ is detailed and rationalizes underlying structure–property relationships in this series of diradicaloids.1
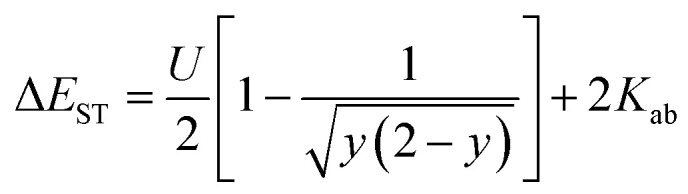
2
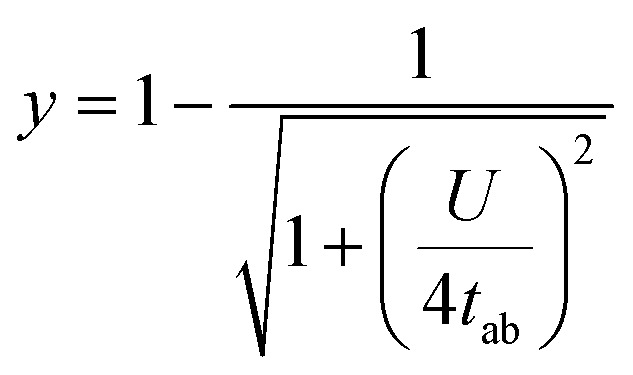


Analysis of DIAn 47 provided a calculated Δ*E*_ST_ value of −4.7 kcal mol^−1^ and serves as a reference point for the remaining derivatives (Table 4). In the case of 48–50, *t*_ab_ appreciably decreases corresponding to an increase in OS character in accordance with eqn (2). *U* only slightly changes between these three isomers highlighting the major contribution of external delocalization to *t*_ab_. DIAn 51 has the smallest *t*_ab_ of this set and a larger *U* attributed to the electron rich S-atom enhancing diradical character with both parameters. Despite the trend in increasing *y*_0_, predicted Δ*E*_ST_ values hold 48 and 50 as the largest and smallest gaps, respectively, in this set of diradicaloids. DIAn 52 increases in its calculated Δ*E*_ST_ to −5.4 kcal mol^−1^ with slightly higher coulombic repulsions and transfer integral values relative to 47 (Table 4). TIPSethynyl substitution therefore assists in stabilizing the triplet state in all DIAns to some degree. This computational analysis illustrates the balance between *U* and *t*_ab_ on both diradical character and Δ*E*_ST_, where they trend proportionally together; however, careful consideration of heterocycle fusion is needed as in the case of 51.

### SQUID magnetometry

**Fig. 14:**
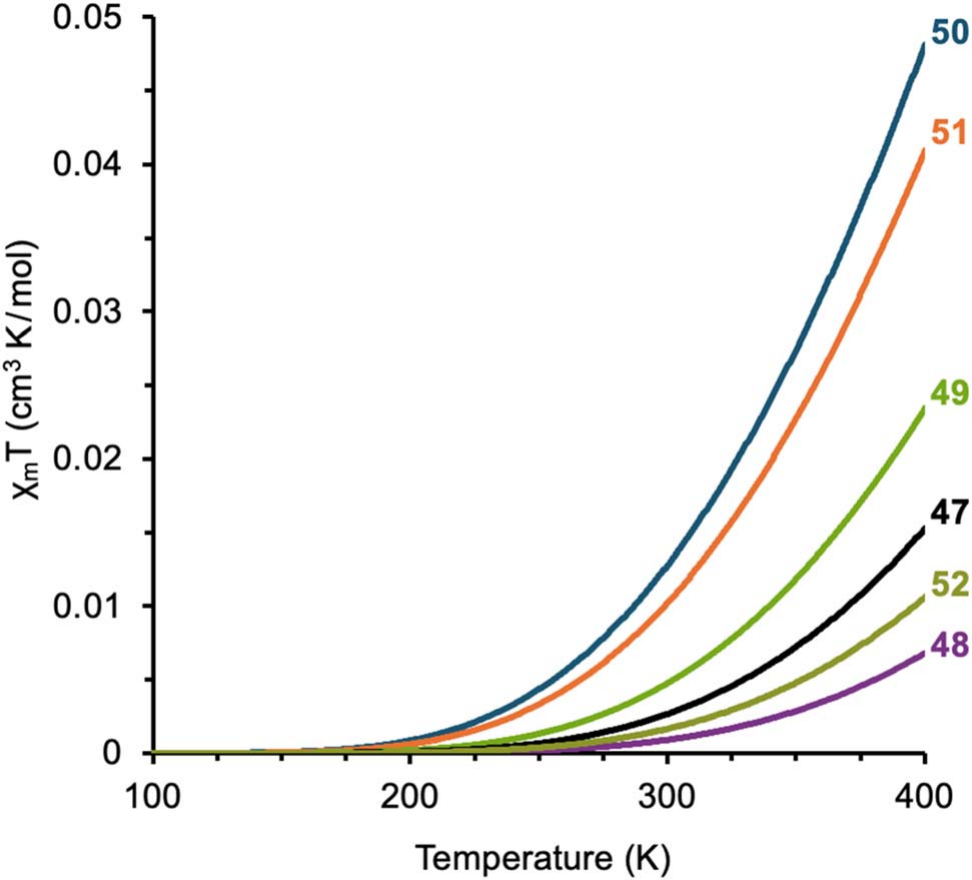
Bleaney–Bowers fits of heating cycle SQUID data for the DIAn series 48–52; whereas heating of 48–51 stopped at 400 K, the trace for 52 is truncated at 400 K for consistency.

A crucial part of the puzzle is experimental determination of Δ*E*_ST_ by superconducting quantum interference device (SQUID) magnetometry. This technique was initially employed on 47 by the Gómez-García group from the University of Valencia for experimental measure of Δ*E*_ST_ where small magnetic responses were recorded while heating powder samples to 400 K. Having fit the collected data to the Bleaney–Bowers equation (Fig. 14),^72^ the experimental Δ*E*_ST_ for 47 was −4.2 kcal mol^−1^, in reasonable agreement with its predicted value. Similar examination of 52 shows a change of +0.4 kcal mol^−1^, agreeing with the predicted increase of the Δ*E*_ST_ compared to 47. SQUID magnetometry performed on 48–51 revealed Δ*E*_ST_ values (Table 4) that are in reasonable agreement with calculated estimates when fit to the Bleaney–Bowers equation and confirm the ordering of Δ*E*_ST_ in DIAns 47–52 where 48 features the largest spin-state energy gap and 50 has the smallest gap. Our diradicaloids exhibit the power of logical structural alterations in fine tuning magnetic behavior (ΔΔ*E*_ST_ = −1.6 kcal mol^−1^ for the series) with greater radical delocalization (smaller *t*_ab_) and minimal coulombic repulsions (smaller *U*) favoring small Δ*E*_ST_ values. This level of control over spin-paired and spin-unpaired states is desirable for magnetic material applications for on-demand transitions.

### Caught in the middle: DCN-based molecules straddle closed- and open-shell structures

**Fig. 15:**
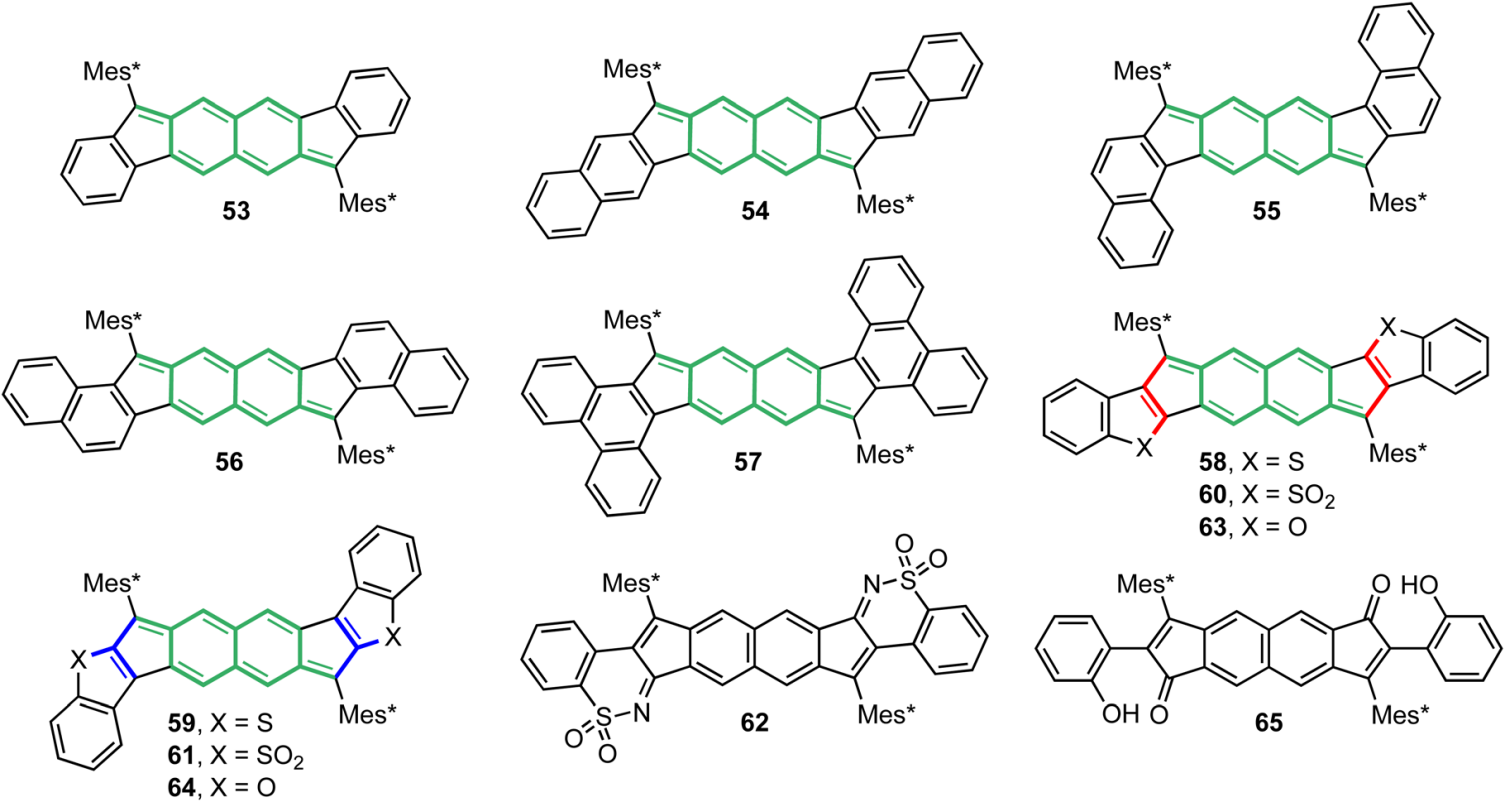
FF homologues 53–61 and 63–64 along with decomposition products 62 and 65. NQD substructures are highlighted while linear and cross conjugation patterns are bolded in heterocycle-fused derivatives according to Fig. 5.

Recalling the continuum from antiaromaticity to diradical character, the sections presented so far in this perspective have illustrated our group's mastery over the two extreme ends where IFs only exhibit CS behavior and DIAns exhibit prominent OS behavior. As suggested by its placement in the continuum, the NQD unit within the DCN core provides the basis for moderate diradicaloids and would help elucidate the cross-over from CS to OS characteristics. The aforementioned [4,3-*c*]FF had a *y*_0_ value of 0.377 and no observable indication of an OS electronic structure.^67,73^ Efforts for in-depth investigations were then placed on [3,2-*b*]FF 53 (Fig. 15) as it was predicted to have greater OS character (*y*_0_ = 0.434).^73,74^ Optoelectronic properties were indeed enhanced because of longer conjugation with a red-shifted low energy absorption and a very weak low energy shoulder teasing a doubly-excited configuration; however, the lack of peak broadening in the proton NMR spectrum up to 100 °C and distinct bond alternation within the quinoidal core led us to erroneously believe that 53 was purely CS when first reported in 2017.

### Breaking open diradical character in fluorenofluorenes

Strategies for developing diradical character in FFs ultimately followed our theme of external arene modification to push its homologues into the intermediate diradicaloid regime. Angular dibenzo-fusion was adapted to the FF scaffold since π-extension influenced paratropicity and OS character in previous studies. We hypothesized that large changes to *t*_ab_ in FFs would boost diradical character as it did in the DIAn series. Computational analysis supported this hypothesis, as increasing *y*_0_ values evolved over *linear*-, *syn*-, and *anti*-fusion as *t*_ab_ decreased (Table 5).^74^ Further π-extension in 57 by way of phenanthrene fusion pushed the extent of FF OS character even further within the hydrocarbon series (*y*_0_ = 0.629).

**Table 5:** Electronic parameters and singlet–triplet energy gap for FF derivatives 53–61, 63 and 64

Cmpd	*U*/2 (eV)^a^^,^^b^	*t*_ab_ (eV)^a^^,^^b^	*y*_0_ a ^,^ c	Δ*E*_STcalc_ (kcal mol^−1^)^a^^,^^d^	Δ*E*_STexpt_ (kcal mol^−1^)
FF 53	1.446	1.163	0.492	−10.25	−9.3
*linear*-DBFF 54	1.315	1.143	0.512	−10.03	−9.6
*syn*-DBFF 55	1.352	1.055	0.559	−9.63	−8.7
*anti*-DBFF 56	1.336	1.012	0.595	−8.61	−7.8
TBFF 57	1.349	0.96	0.629	−7.90	−7.6
*anti-*IIDBT 58	1.563	1.031	0.613	−8.77	−8.1
*syn*-IIDBT 59	1.404	0.905	0.658	−8.06	−7.2
*anti*-IIDBTS 60^d^	—	—	0.601	−9.65	—
*syn*-IIDBTS 61^d^	—	—	0.652	−8.29	−6.5
*anti*-IIDBF 63	1.677	1.058	0.623	−9.10	—
*syn*-IIDBF 64	1.38	0.865	0.682	−7.68	−6.0

a Geometries optimized at the R- and UB3LYP/6-311G* levels.

b Estimated at the CASCI(2,2)6-311G* level using the (tuned-)LC-RBLYP MOs (denoted as tuned-LC-RBLYP-CASCI(2,2)/6-311G*).

c Calculated at the PUHF/6-311G* level.

d Adiabatic Δ*E*_ST_ value calculated at the spin-flip noncollinear (SF-NC)-TDDFT PBE5050/6-311G* level along with R- or UB3LYP/6-311G* zero-point vibrational energy correction for each spin state. Electronic parameters were not calculated for these entries.

**Fig. 16:**
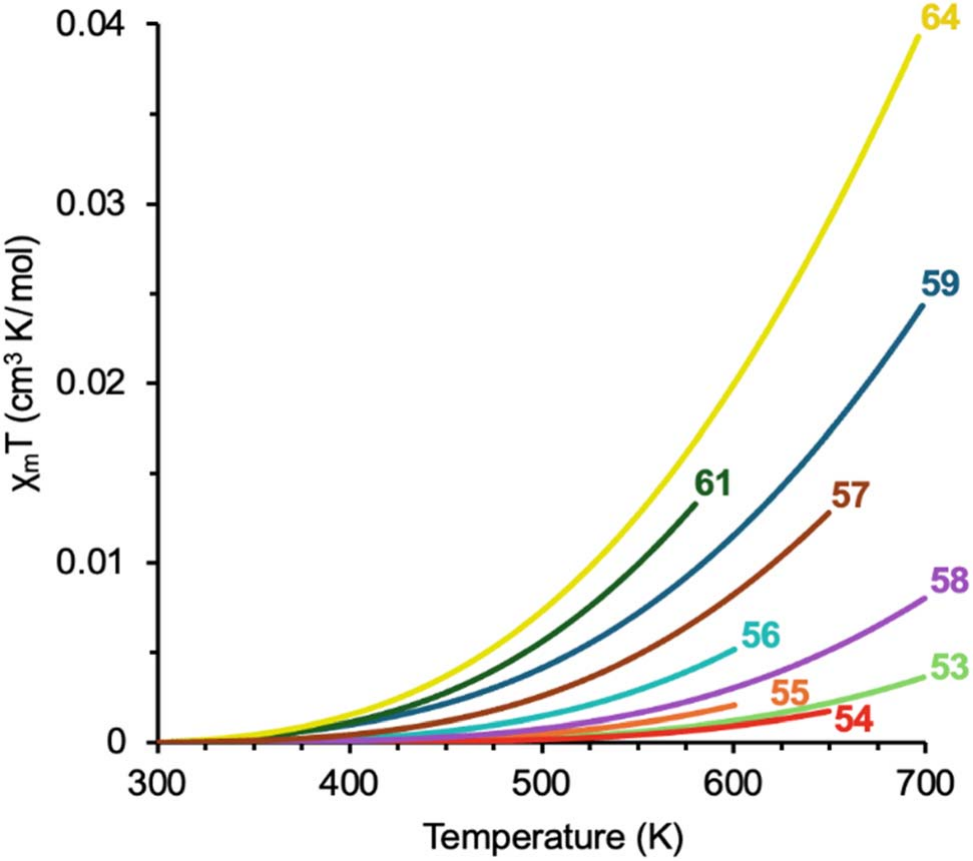
Bleaney–Bowers fits of recorded SQUID data for FF homologues 53–59, 61, and 64.

In collaboration with the Kato group at the University of Shiga Prefecture, FFs 54-57 were synthesized following the “inside-out” II strategy. Characterization of this new set of dibenzofluorenofluorenes (DBFFs) confirmed appreciable diradical character in 56 and 57 with peak broadening in the ^1^H NMR spectra at elevated temperatures (∼90 °C) and double exciton state signatures in the NIR region. *syn*-DBFF 55 showed weak indications of OS character requiring ∼150 °C to observe minimal ^1^H signal broadening and 54 exhibited a CS structure like 53. Fortunately, an upgrade to the SQUID magnetometer at the University of Valencia now permitted access to temperatures up to 800 K; thus, experimental data for the weaker diradicaloids could be obtained.§§The maximum SQUID temperature initially was 400 K when 47 was examined. The instrument was upgraded in 2017 and can achieve temperatures upwards of 800 K. Since then, the standard protocol has been to acquire both DSC and TGA data on our samples and then stop SQUID data acquisition 25 K before the onset of decomposition. In addition, a SQUID cooling curve is also acquired to corroborate the heating data and to show that no covalent modification of the framework has occurred. The spin-state dynamics of these FFs illustrate stepwise decrease in Δ*E*_ST_ from 54→53→55–57 (Fig. 16). Like in the DIAn series, linear 54 has a larger Δ*E*_ST_ compared to parent 53 due to the slight increase in U corresponding to a greater electronic screening between the peripheral arene moiety and the core.

Heterocycle fusion in 51 is known to affect dramatically the electronic parameter *U*, contributing to elevated diradical character while widening Δ*E*_ST_ relative to 50 on account of Pauli repulsions disfavoring the unpaired spin-state. Fusion of DCN with benzothiophene, like in 51, can result in an *anti*-relationship between the S-atom and apical carbon of the core's five-membered ring. The *syn*-relationship can also be attained when reversing the fusion mode of benzothiophene onto DCN. As such, electron-rich indenoindenodibenzothiophenes (IIDBTs) 58 and 59 were prepared by Justin Dressler and Joshua Barker, respectively.^75^ These isomers feature different conjugation patterns between the radical center and S-atom where the *anti*-isomer has a linear pattern, and the *syn*-isomer has a cross pattern (Fig. 5). Following these conjugation patterns, there is less interaction between the unpaired electrons and S-atoms in 59 as they come within two atoms from each other while they can neighbor each other in 58. Applying the same late-stage modification used to prepare sulfones 27 and 28 should give the IIDBTS analogues, yielding changes to their OS characteristics as we know the S-atom influences both *y*_0_ and Δ*E*_ST_. Oxidation of 59 furnished sulfone 61, whose calculated *y*_0_ and Δ*E*_ST_ values show that it retains the diradical character of 59 yet it is expected to exhibit an earlier spin-state transition than 60.^50^ Unfortunately, oxidation of 58 yielded decomposition product 62 (X-ray) and not sulfone 60.^50^

Relating these structural considerations to *U* and *t*_ab_, both parameters are greater in 58 than they are in 59, as listed in Table 5, leading to increased OS character and a slightly narrower singlet–triplet gap for the *syn*-isomer. This is supported by the slight elongation of the bond between the apical carbon and the core (1.419 and 1.421 Å, respectively) and red-shifting of low energy absorption features from *anti*-to *syn*-fusion. The key evidence lies in SQUID results confirming the narrowed Δ*E*_ST_ in 59, relative to 58. When comparing these heterocycle-fused isomers to the hydrocarbon series, 56 and 57 sit between 58 and 59 in the range of Δ*E*_ST_ values for FF derivatives. Importantly, the ordering of FF derivatives 53–56 and 58 mirrors the ordering of DIAns 47–51, highlighting the consistent effect of electron repulsions with linear conjugation between the radical carbon and S-atom across multiple analogues. IIDBT 59 capitalizes on the balance between π delocalization and coulombic repulsions and offers a design strategy to enhance *y*_0_ and Δ*E*_ST_ through *syn*-fusion. The experimentally determined Δ*E*_ST_ of 61 subverts that of 59, which is a sensible discovery since oxidation of these S-atoms imparts electron deficiency, further decreasing electronic repulsions near the cross-conjugated pathway.

Exploration of heteroatom effects on diradical character in the FF scaffold continued with the inclusion of benzofurans as the O-atoms would provide even greater electronegativity, which we know enhanced molecular properties in the IF congeners 29–30.^52^ Quantum chemical calculations on indenoindenodibenzofurans (IIDBFs) 63–64 painted these regioisomers as worthwhile targets on account of increased OS character compared to their benzothiophene counterparts. Calculated Δ*E*_ST_ values suggested *syn*-IIDBF 64 to be more magnetically interesting between the two, as we would now expect given the behavior of 59 compared to 58. Unfortunately, all attempts to prepare 63 instead afforded ring-opened product 65, analogous to the case of 30, where ring-opened material predominated.^52^*syn*-IIDBF 64 was remarkably stable and its high diradical character with SQUID data revealed that 64 has the smallest Δ*E*_ST_ of the FF homologues at −6.0 kcal mol^−1^. Such results exemplify the profound effect electronegativity has on optoelectronic and magnetic properties in these highly conjugated systems. Looking at the ground covered by our forays into diradicaloids shows a wide window of the Δ*E*_ST_ landscape filled by our lab comprising a total range of 6.4 kcal mol^−1^ across the DIAn and FF families, from as low as −3.2 to as high as −9.6 kcal mol^−1^. Such efforts effectively demonstrate our mastery of tuning OS character and Δ*E*_ST_ through our judicious molecular designs.

### What IF we turned to devices?

Having produced a large library of diarenoindacenes and diindenoarenes, we have covered a wide range of frontier orbital energy levels, low energy absorptions, and redox potentials through precise structural modification. The (mostly) CS nature of IF derivatives allows replacement of the bulky Mes and Mes* groups on their apical carbons with (trialkylsilyl)ethynyl groups, which are well-known to provide favorable solid-state packing and solution processability in acenes.^40,76^ These properties are desirable for semiconductor applications, as was mentioned several times throughout this perspective, so how do these molecules perform in device active layers in OFETs?

**Fig. 17:**
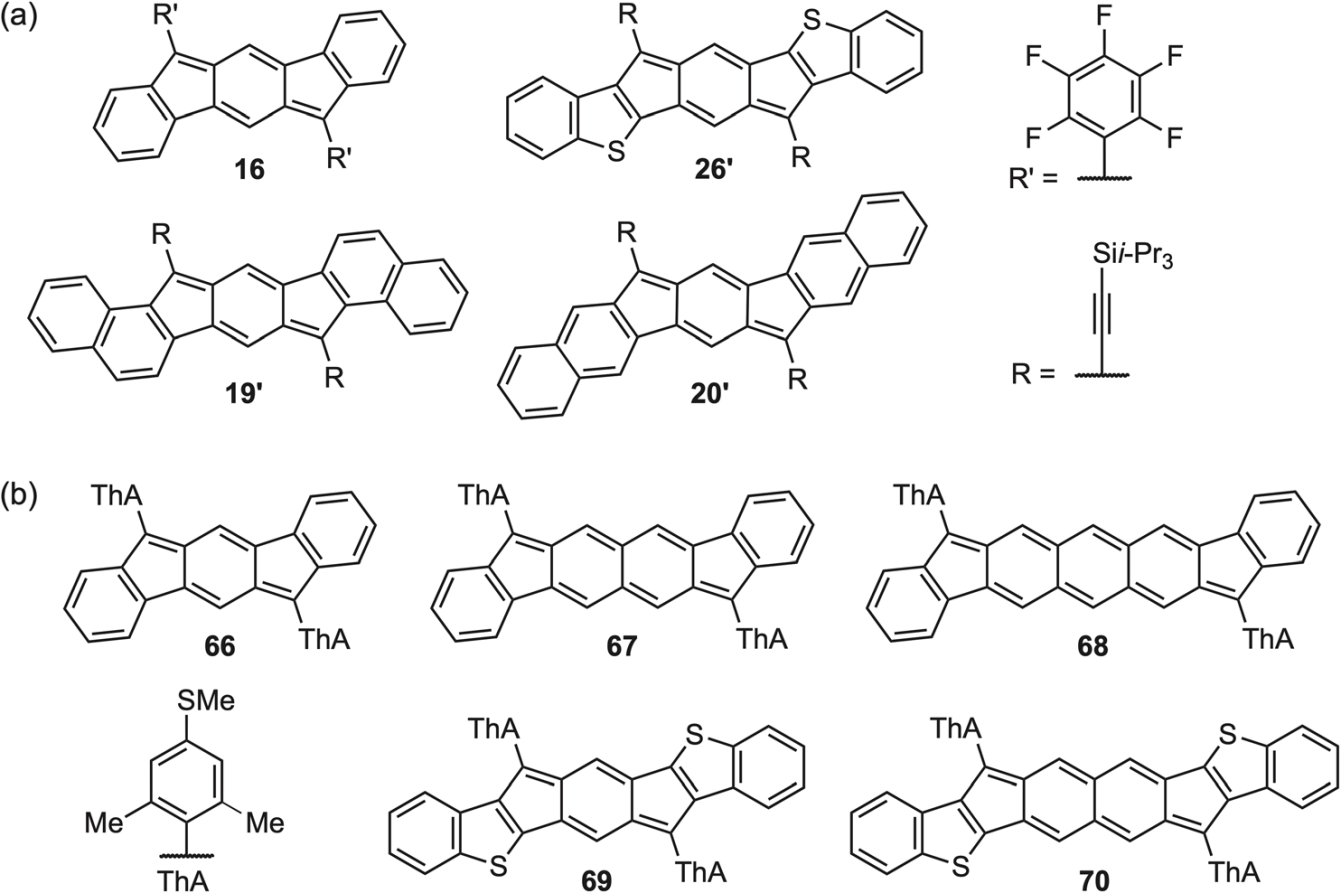
Chemical structures of quinoidal derivatives used in (a) OFET evaluation and (b) single molecule conductance measurements.

1. Our first foray in 2012 examined single crystal OFETs of perfluorophenyl-substituted [1,2-*b*]IF 16 (Fig. 17a, R’ = C_6_F_5_), which yielded weak and modestly balanced ambipolar transport with hole and electron mobilities of 7 × 10^−4^ and 3 × 10^−3^ cm^2^ V^−1^ s^−1^, respectively.^42^ While the mobility values were low, this result was an important proof-of-concept for our new organic semiconductor scaffold.

2. Ambipolar behavior was observed in 2016 in OFETs containing thin films of DIAn 47, which showed hole and electron mobilities of 2 × 10^−3^ and 4 × 10^−3^ cm^2^ V^−1^ s^−1^, respectively.^68^ The balanced values seen here are ideal for effective ambipolar transport, but performance was limited due to 1D stacking of 47 in the solid state.

3. Switching to (triisopropylsilyl)ethynyl groups in *anti*-IDBT 26′ resulted in significantly improved hole-carrier transport in an OFET (0.44 cm^2^ V^−1^ s^−1^), but unfortunately we did not study electron-carrier transport.^49^ Shortly after our work, a group in China showed that 26′ was indeed ambipolar, with hole mobilities up to 0.64 cm^2^ V^−1^ s^−1^ and an electron mobilities up to 0.34 cm^2^ V^−1^ s^−1^.^77^

4. Our best OFET devices used spin-coated layers of TIPSethynyl-substituted *linear*- and *anti*-DNI regioisomers 20′ and 19′ (Fig. 17a) which were vapor annealed and retained their 2D bricklayer packing. Evaluation of charge-carrier transport revealed average hole mobilities of 1.04 cm^2^ V^−1^ s^−1^ and 4.72 cm^2^ V^−1^ s^−1^ for TIPS linear-DNI and TIPS *anti*-DNI, respectively, with some values as high as 7 cm^2^ V^−1^ s^−1^.^78^

Unfortunately, engineering the solid-state packing of OS molecules with simpler/smaller functional groups remains limited by the reactivity at the radical centers, as demonstrated during hydrogen/deuterium abstraction experiments with 58,^75*a*^ as well as by the dimeric structure shown in Fig. 11.

Another method to probe transport properties that circumvents poor packing is scanning tunneling microscope-break junction (STMBJ) technique.^79^ Key molecular handles needed to bind to Au electrodes in this technique are Lewis bases, such as thioethers in our case, which were incorporated into a select group of our molecules (66–70, Fig. 17b) to undergo room temperature single molecule conductance experiments. In collaboration with the Kamanetska group at Boston University, we achieved conductance enhancements over two orders of magnitude (*G*_0_ = 10^−4^–10^−2^) in single molecule circuits formed with diradicaloids 66–68 upon increasing molecular length by ∼5 Å.^80^ This large, atypical anti-ohmic conductance enhancement at longer molecular lengths is attributed to the diradical character of the molecules, which results in constructive interference between the frontier molecular orbitals, allowing for robust and facile measurements of their transport properties. Even stronger anti-ohmic behavior was observed in 69 and 70, which suggests that this class of quinoidal/diradicaloid materials includes promising candidates for creating highly conductive and tunable nanoscale wires.

Future endeavors in IF based single-molecule junctions can shift towards spin filter experiments,^81*a*^ particularly with derivatives featuring readily accessible triplet states at room temperature (*e.g.*, 49–51) to capitalize on parallel spins. Such experiments will probe the suitability of IF diradicaloids as single-molecule gates and contribute to the greater effort towards organic spin filters.^81^ A true pivot for IFs can look towards photothermal applications given the strong solution state low-energy absorption observed across these non-radiative compounds.^48,82^

## Conclusions

Over the last 15 years, the Haley lab has contributed a significant library of molecules to the antiaromaticity and diradicaloid literature. There have been several synthetic routes presented in our work and their modularity provides a powerful tool to logically alter and extend conjugation in both the core and peripheral-fused arenes. Heterocycle fusion adds additional possibilities as synthetic targets, as we have shown with benzothiophene, benzofuran, and indole. Our synthetic toolbox has allowed us to construct molecules where we systematically alter molecular properties in a controlled, rational manner, thus providing important structural insights on how to manipulate the interrelated topics of antiaromaticity and diradical character. We believe these same insights will allow us to design and assemble larger structures that contain significant tetraradicaloid (and even polyradical) character.^83^ While some inroads have been made into preparing indenofluorene-based tetraradicaloids,^31*a*,84^ the minimal tetraradical character (*y*_1_ < 0.1) calculated for these structures meant that evidence of any tetraradicaloid properties was elusive; thus, there remains significant room for improvement. By judicious choice of the building blocks that we have previously demonstrated to impart open-shell characteristics, *i.e.*, structural refinement, we are optimistic that we can crack open the tetraradicaloid nut.

While we have certainly made our mark with these achievements, it is no secret that there are many other players in the antiaromatic/diradicaloid arena. This field remains highly active with major contenders such as the Tobe group producing fluoreno[2,3-*b*]fluorene^65,85^ and indeno[2,1-*b*]fluorene,^27^ the Stepien group presenting diindeno[1,2-*a*:2′,1′-*i*]phenanthrene^86^ and twisted alkene oligoradicaloids,^87^ the Wu group with periacenes^88^ and zethrenes,^89^ the Chi group reporting nanographene fragments^90^ and heteroacenes,^91^ and many others.^92^ New minds are also making impacts with indenofluorene topologies for materials applications from Millán's group^93^ and with even larger conjugated systems incorporating multiple *s*- and *as*-indacene moieties like those from the Das group.^31^ We hope that our myriad contributions offer inspiration to emerging groups and motivation to continue research in antiaromatic, OS molecules, and their device performance as the desire for novel organic electronic materials persists.

## Author contributions

J. E. B. and G. I. W. wrote the initial draft manuscript. E. V. and M. M. H. revised and expanded upon this work. All authors contributed to the editing of the final draft. All authors have approved the final version of the manuscript.

## Conflicts of interest

There are no conflicts to declare.

## Data Availability

The only new data reported in this article are the previously unpublished X-ray structures shown in Fig. 11. All other data can be found in the primary literature cited. CCDC 2416145 and 2416146 contain the supplementary crystallographic data for this paper.^94*a,b*^
